# Regulatory mechanisms of fruit ripening: an insight into DNA, histone, and RNA modifications

**DOI:** 10.1093/hr/uhag235

**Published:** 2026-05-20

**Authors:** Tingkai Zhai, Chenwen Zhou, Fei Xie, Yudie Chen, Jiayi Chen, Zimeng Yang, Sijia Yang, Honglu Hu, Shaojia Li, Jinping Cao, Yue Wang, Chongde Sun

**Affiliations:** Laboratory of Fruit Quality Biology, The State Agriculture Ministry Laboratory of Horticultural Plant Growth, Development and Quality Improvement, Zhejiang Provincial Key Laboratory of Integrative Biology of Horticultural Plants, Zhejiang University, Hangzhou 310058, China; Hainan Institute, Zhejiang University, Sanya 572025, China; Laboratory of Fruit Quality Biology, The State Agriculture Ministry Laboratory of Horticultural Plant Growth, Development and Quality Improvement, Zhejiang Provincial Key Laboratory of Integrative Biology of Horticultural Plants, Zhejiang University, Hangzhou 310058, China; Laboratory of Fruit Quality Biology, The State Agriculture Ministry Laboratory of Horticultural Plant Growth, Development and Quality Improvement, Zhejiang Provincial Key Laboratory of Integrative Biology of Horticultural Plants, Zhejiang University, Hangzhou 310058, China; Laboratory of Fruit Quality Biology, The State Agriculture Ministry Laboratory of Horticultural Plant Growth, Development and Quality Improvement, Zhejiang Provincial Key Laboratory of Integrative Biology of Horticultural Plants, Zhejiang University, Hangzhou 310058, China; Laboratory of Fruit Quality Biology, The State Agriculture Ministry Laboratory of Horticultural Plant Growth, Development and Quality Improvement, Zhejiang Provincial Key Laboratory of Integrative Biology of Horticultural Plants, Zhejiang University, Hangzhou 310058, China; Laboratory of Fruit Quality Biology, The State Agriculture Ministry Laboratory of Horticultural Plant Growth, Development and Quality Improvement, Zhejiang Provincial Key Laboratory of Integrative Biology of Horticultural Plants, Zhejiang University, Hangzhou 310058, China; Laboratory of Fruit Quality Biology, The State Agriculture Ministry Laboratory of Horticultural Plant Growth, Development and Quality Improvement, Zhejiang Provincial Key Laboratory of Integrative Biology of Horticultural Plants, Zhejiang University, Hangzhou 310058, China; Laboratory of Fruit Quality Biology, The State Agriculture Ministry Laboratory of Horticultural Plant Growth, Development and Quality Improvement, Zhejiang Provincial Key Laboratory of Integrative Biology of Horticultural Plants, Zhejiang University, Hangzhou 310058, China; Laboratory of Fruit Quality Biology, The State Agriculture Ministry Laboratory of Horticultural Plant Growth, Development and Quality Improvement, Zhejiang Provincial Key Laboratory of Integrative Biology of Horticultural Plants, Zhejiang University, Hangzhou 310058, China; Laboratory of Fruit Quality Biology, The State Agriculture Ministry Laboratory of Horticultural Plant Growth, Development and Quality Improvement, Zhejiang Provincial Key Laboratory of Integrative Biology of Horticultural Plants, Zhejiang University, Hangzhou 310058, China; Laboratory of Fruit Quality Biology, The State Agriculture Ministry Laboratory of Horticultural Plant Growth, Development and Quality Improvement, Zhejiang Provincial Key Laboratory of Integrative Biology of Horticultural Plants, Zhejiang University, Hangzhou 310058, China; Laboratory of Fruit Quality Biology, The State Agriculture Ministry Laboratory of Horticultural Plant Growth, Development and Quality Improvement, Zhejiang Provincial Key Laboratory of Integrative Biology of Horticultural Plants, Zhejiang University, Hangzhou 310058, China; Hainan Institute, Zhejiang University, Sanya 572025, China

## Abstract

Complex regulatory networks formed by epigenetic modifications such as DNA, histones, and RNA can influence gene expression without altering DNA sequences, participating in ripening processes and quality changes in fruits. Although significant progress has been made in understanding epigenetic modifications and their roles in fruit ripening, the regulatory network underlying these complex relationships remains incompletely elucidated. Here, we summarize the key molecular regulatory networks involving DNA, histone, and RNA modifications in fruit ripening and secondary metabolite changes in recent years; review the research applications of epigenetic modification inhibitors and omics technologies, elucidating their advantages and limitations; and ultimately propose a comprehensive strategic model for future studies on epigenetic modification regulation of fruit ripening. These findings not only deepen our understanding of epigenetic modifications during fruit ripening but also raise new questions and research directions. It provides a deeper understanding of the ripening process and quality changes in fruits, offering insights for new strategies in fruit production and integrated regulation.

## Introduction

The transition to sustainable diets is one of the most critical global challenges of the twenty-first century. This implies an increase in the demand for horticultural plants, particularly fruit and vegetables, to replace traditional dietary patterns [1, 2]. Moreover, with societal development and progress, people’s demand for these horticultural plants has also begun to shift, particularly for fruits. The demand has transitioned from ‘accessibility’ to ‘high quality’ and ‘health benefits’ [3–5]. To meet market demands, systematic research has been conducted on the ripening process and quality changes of fruits. Integrated regulatory strategies encompassing fruit ripening and development [6], postharvest storage [7], processing, and transportation [8] have been developed as a result. Fruit ripening is a complex process that directly affects postharvest yield and quality. Horticultural plants primarily feature fleshy fruits, which can be classified into two types based on their respiratory activity in the early stages of ripening: climacteric fruits and nonclimacteric fruits. Climacteric fruits utilize ethylene as the core signal, exhibiting significantly enhanced respiration during early ripening [9], followed by changes in colour, aroma, texture, and nutrient content [10]. In contrast, nonclimacteric fruits are regulated by multiple hormones, including abscisic acid (ABA), jasmonic acid (JA), and auxin (IAA), showing minimal respiratory changes during early ripening [11, 12]. It is also primarily related to the fact that hormones can influence the expression of genes associated with fruit ripening through transcriptional and post-translational modification (PTM) regulation. In addition, fruit ripening is also influenced by epigenetic modifications [6, 13].

In eukaryotes, epigenetic modifications carry heritable regulatory information without altering the DNA sequence. To explain the phenomenon where biological phenotypes exhibit marked differences despite unchanged DNA sequences, scientists proposed a concept called ‘epigenetics’ in 1942. This is a combination of ‘genetics’ and ‘epigenesis’ [14]. After almost 100 years, a key regulatory network has been revealed, including epigenetic modifications like DNA methylation, histone modifications, RNA modifications, changes to the structure of chromosomes, and the organization of chromosomes into domains (TADs) [15]. In plants, studies of epigenetic modifications have been conducted in model plants like *Arabidopsis thaliana* and major crops such as *Oryza sativa*, *Zea mays*, and *Triticum aestivum* [16]. Research areas include DNA methylation [17], histone modifications [18], histone variants [19], chromatin remodeling factors [20], noncoding RNAs [21], RNA modifications [22], and chromatin higher-order structure [23]. These epigenetic modification studies also help address biological questions related to embryonic development [24], plant regeneration [25], vernalization [26], plant architecture regulation [27], nodulation and nitrogen fixation [21], biotic/abiotic stress [28, 29], and hybrid vigor [30]. Research on epigenetic modifications in horticultural plants has passed the exploratory stage and entered a period of rapid development. In addition to the extensive research of epigenetic modifications in other plant species, recent years have seen analyses of developmental, ripening, and secondary metabolite processes in horticultural plants. Research areas include DNA methylation and active demethylation [31], histone modifications [32], and RNA modifications [33]. Among these, climacteric fruits *Solanum lycopersicum* and nonclimacteric fruits strawberry (*Fragaria* × *ananassa* and *Fragaria vesca*) have been the most extensively studied as classic models. Their research has driven advances in the understanding of epigenetic molecular regulatory mechanisms in fruit ripening and quality changes. Previous studies have provided detailed and accurate reviews of epigenetic modifications during fruit ripening and quality changes. These studies have advanced our understanding of fruit maturation and its underlying regulatory mechanisms [31–35]. However, the specific molecular regulatory networks involved remain poorly defined, and the potential applications of these modifications in production have yet to be fully explored. In this review, we systematically mapped the regulatory networks of key research advances in DNA, histone, and RNA modifications during fruit ripening in recent years; summarized the effects of exogenous inhibitors associated with epigenetic modifications, and reviewed the application of various omics technologies. This review aims to establish an integrated research model for epigenetic modifications that combines omics technology applications, molecular regulation analysis, and exogenous substance regulation. This approach will provide a new insight and understanding of strategies for the comprehensive regulation of fruit ripening and quality changes.

## DNA methylation and active demethylation

DNA methylation is widely present in eukaryotes and represents a conserved epigenetic mark. It primarily involves two types: the conversion of adenine to *N^6^*-methyladenine (6mA) and the conversion of cytosine to *C^5^*-methylcytosine (5mC) [36]. In plants, current research primarily focuses on 5mC DNA methylation. In fact, 5mC levels in plants are typically higher than those in animals, and the rate of cytosine methylation can be up to 50% [37]. However, only in *A. thaliana* [38], *O. sativa* [39], and *Z. mays* [40] has 6mA methylation been found to play a crucial role in biological functions such as seed formation, vegetative growth, photosynthesis, and the response to heat stress. The primary sites of DNA methylation in plants are symmetric CG, CHG, and asymmetric CHH. CG and CHG methylation simultaneously modify the corresponding cytosines in double-stranded DNA, whereas CHH methylation occurs only in single-stranded DNA [16]. Among these, chromatin methyltransferases (CMTs) that maintain CHG methylation are only found in plants. This result means that CHG methylation is much more common in plants than animals [41]. Extensive research has confirmed that *de novo* methylation in plants occurs via the RNA-directed DNA methylation (RdDM) pathway [42]. Firstly, double-stranded RNA (dsRNA) synthesized by RNA-dependent RNA polymerase 2 (RDR2) employing Pol IV transcripts as templates is processed by Dicer-like 3 (DCL3) to yield 24-nt small interfering RNA (siRNA). This siRNA is then loaded onto Argonaut 4 (AGO4), which facilitates its entry into the nucleus. Domains rearranged methyltransferase 2 (DRM2) acts in concert with AGO4 to establish DNA methylation of the Pol V transcription template strand [43–45]. Based on this RdDM pathway prevalent in plants, we mapped the DNA modification regulatory network during fruit ripening and distinguished differences between climacteric fruits and nonclimacteric fruits within the regulatory network (Fig. 1).

**Figure 1:**
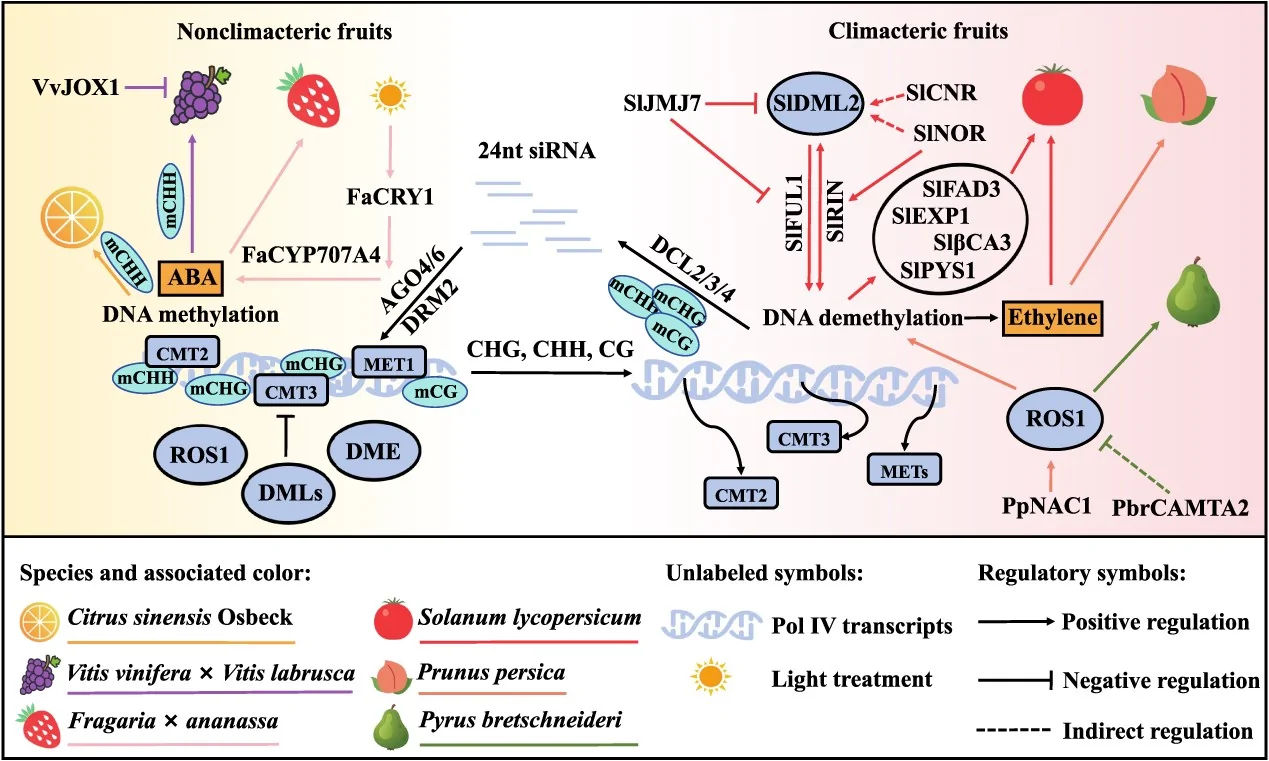
Regulation of RdDM pathway during fruit ripening, including both nonclimacteric fruits and climacteric fruits. Nonclimacteric fruits: strawberry (*Fragaria* × *ananassa*), grape (*V. vinifera* × *V. labrusca*), citrus (*C. sinensis* Osbeck); climacteric fruits: tomato (*S. lycopersicum*), peach (*P. persica*), and pear (*P. bretschneideri*). Nonclimacteric fruits primarily promote ripening through DNA methylation, which might be associated with high levels of mCHH methylation and the ABA signaling pathway. Climacteric fruits primarily promote ripening through active DNA demethylation mediated by ROS1 and DML2; it is primarily linked to the impact of ethylene biosynthesis. Positive regulation is indicated by arrows, while negative regulation is represented by T-shaped symbols.

High levels of DNA methylation promote the ripening of nonclimacteric fruits. The maintenance of DNA methylation in plants aids the restoration of 5mC modifications in newly formed DNA strands, primarily through the action of Methyltransferase 1 (MET1) and Chromomethylase 2/3 (CMT2/3) [46]. Among these, MET1 primarily interacts with mCG-binding proteins (VIM1/2/3) to maintain mCG methylation through a semiconservative mechanism [46]. CMT2 and CMT3 share a similar catalytic mechanism, and the difference is that CMT2 primarily maintains mCHH methylation in heterochromatic regions, whereas CMT3 positively regulates mCHG methylation levels [47]. During the ripening of nonclimacteric fruits, higher levels of DNA methylation are observed. This increasing trend may represent an interplay between environmental factors, plant hormones, and epigenetic modifications. In addition, mCHH may be a key site that positively regulates DNA methylation levels in nonclimacteric fruits. Firstly, the ripening of nonclimacteric fruits is influenced by external environmental factors, such as light and temperature. DNA methylation can act as a mechanism to respond to environmental signals, which enables fruits to adjust their ripening process in response to changes in external conditions. For example, a study of *Fragaria* × *ananassa* revealed that bagged fruits exhibit delayed ripening and significantly reduced DNA methylation levels in the promoter region of ABA 8′-hydroxylase (*FaCYP707A4*), a gene derived from *F. vesca* [48], and suppressed expression of blue light receptor *CRYPTOCHROME 1* (*FaCRY1*). When exposed to light, *FaCRY1* interacted with FaAGO4, which enhanced methylation levels by binding to the *FaCYP707A4* promoter region and promoted fruit ripening [49]. Secondly, the ripening of nonclimacteric fruits is regulated by multiple hormones. These hormonal signaling pathways are crucial for DNA methylation, especially in response to changes in ABA levels. The exogenous application of 500 ppm ABA +0.1% Tween 20 to *Vitis vinifera* significantly promoted fruit ripening. Differentially expressed genes (DEGs) are enriched in defense-related metabolism, chlorophyll degradation processes, and ripening-related metabolic pathways, accompanied by genome-wide high levels of DNA methylation [50]. High methylation in *Citrus sinensis* Osbeck was highly correlated with the active expression of hundreds of genes, primarily involved in fruit ripening and mainly participating in the ABA hormone signalling pathway [51]. The bagging treatment reduced DNA methylation levels of *FaCYP707A4* in *Fragaria* × *ananassa*, which was also associated with reduced endogenous ABA accumulation [49]. Furthermore, significant changes in mCHH might primarily influence DNA methylation levels in nonclimacteric fruits. For example, the early-ripening bud sport variety ‘Fengzao’ exhibited higher methylation levels in transposons and gene-flanking regions than ‘Kyoho’ in *V. vinifera*, primarily due to the regulation of CHH site hypermethylation mediated by JASMONIC ACID OXIDASE 1 (VvJOX1) [52]. Similar findings were observed in *V. vinifera* varieties ‘Wink’ and ‘Cabernet Savior’, where global genome-wide DNA hypermethylation occurred during fruit ripening, primarily characterized by elevated methylation levels in CHH sequence contexts, particularly in promoter and transposon regions [53]. During the ripening process of *C. sinensis* Osbeck fruit, increased mCHH methylation levels led to elevated genome-wide DNA methylation levels, which are significantly higher than the increases observed in mCG and mCHG methylation levels [51]. However, high levels of whole-genome DNA methylation do not necessarily occur during the ripening process of nonclimacteric fruits. The study in *Fragaria* × *ananassa* revealed that DNA methylation generally decreased during fruit ripening, similar to the climacteric fruit *S. lycopersicum*. This was not driven by the regulation of DNA demethylase genes, unlike the case in *S. lycopersicum*, but rather by the downregulation of genes involved in maintaining DNA methylation [54]. Genome-wide DNA methylation levels in *Fragaria* × *ananassa* have been found to be similarly up-regulated in dark conditions, which corresponds to delayed ripening processes [49]. This suggests that the ripening of nonclimacteric fruits does not rely solely on genome-wide DNA methylation but actually involves a systematic and complex interplay between environmental factors, hormones, gene expression, and epigenetic modifications.

DNA demethylation contributes to faster ripening of climacteric fruits. There are two pathways through which DNA demethylation can occur: one is passive demethylation, which is replication-dependent and involves the gradual dilution of DNA methylation due to its failure to be maintained. The other pathway is active demethylation, which is nonreplication-dependent and requires enzyme catalysis [55]. This represents a crucial dynamic equilibrium process within living organisms. In plants, active demethylation is primarily regulated by the combined action of Repressor of Silencing 1 (ROS1), Demeter (DME), and Demeter-like proteins (DML), which replace 5mC with unmethylated cytosine [37, 56, 57]. Unlike nonclimacteric fruits, climacteric fruits undergo active DNA demethylation during ripening, which is primarily influenced by ethylene signalling. For example, during the ripening process of *Prunus persica*, the transcription level of NAC domain–containing protein 1 (PpNAC1) increased, directly activates the expression of multiple ethylene signalling–associated genes and initiated the process of active DNA demethylation through PpDML1 (also known as PpROS1) [58]. Similar findings were observed in *S. lycopersicum*, where active DNA demethylation positively regulates fruit ripening by controlling ethylene biosynthesis and carotenoid accumulation [59, 60]. At the molecular regulatory level, DML2 and ROS1 play a key role in active DNA demethylation in climacteric fruits. The role of DML2 in demethylation during the ripening and quality development of *S. lycopersicum* fruit was considered a landmark discovery [31]. Specifically, SlDML2 serves as a pivotal regulator of active demethylation during fruit ripening [61, 62], positively regulating Ripening Inhibitor (SlRIN) and FRUITFULL 1 (SlFUL1) to influence chromatin accessibility [59]. During this process, JUMONJI DOMAIN-CONTAINING PROTEIN 7 (SlJMJ7) negatively regulates SlDML2 and SlFUL1 by suppressing the expression of ethylene biosynthetic and ripening-related genes via Histone H3 lysine 4 trimethylation (H3K4me3) [60]. NONRIPENING (SlNOR) positively regulates SlDML2 and SlRIN [61]. SlbCA3 and SlFAD3, which encode β-carbonic anhydrase and ω-3 fatty acid desaturase respectively, are activated at the transcriptional level through SlDML2-mediated DNA demethylation. Both genes participate in the regulation of ripening and disease resistance in fruit [63]. However, when SlRIN expression was restored in *Sldml2* mutants (in which both *SlRIN* and *SlFUL1* were silenced), the expression of more than half of DEGs failed to recover. These results suggest the presence of additional unknown factors downstream of SlDML2 that regulate fruit ripening [59]. Additionally, recent studies have revealed that ROS1 plays a crucial role in climacteric fruits. Analysis of 41 germplasm resources showed that *Pyrus bretschneideri* exhibited an overall increase in DNA methylation during domestication and improvement. This increase in methylation correlates with downregulation of PbrROS1 and primary positive regulation via CALMODULIN-BINDING TRANSCRIPTION ACTIVATOR 2 (PbrCAMTA2) [64]. High levels of DNA methylation caused a reduction in the accumulation of lutein, β-carotene, and ABA in *P. bretschneideri*, and delayed fruit ripening [65]. Overall, DML2 and ROS1 are key nodes in active demethylation, jointly regulating the ripening process of climacteric fruits via ethylene signalling. However, there may still be unknown regulatory genes downstream of DML2, the precise crosstalk and regulatory relationship between active demethylation and ethylene signalling pathways remain unclear. In fact, genome-wide methylation level analysis has also been completed in *Malus domestica*, but no detailed functional analysis has been performed on differentially methylated genes exhibiting differential methylation levels [66].

Active DNA demethylation positively regulates the biosynthesis of secondary metabolites during fruit ripening. The synthesis and degradation of these secondary metabolites are jointly regulated by internal development cues and various other factors [32]. Unlike the differences in DNA modification between climacteric and nonclimacteric fruits during ripening, both types exhibit active DNA demethylation associated with anthocyanin biosynthesis. This change is primarily mediated by MYB domain proteins (MYB) [66], CHALCONE ISOMERASE (CHI), CHALCONE SYNTHASE (CHS) [67], UDP-glucose flavonoid glycosyltransferase (UFGT) [68], and FLAVANONE 3-HYDROXYLASE (F3H) [69], which regulate DNA demethylation during fruit anthocyanin biosynthesis [70]. Furthermore, a study of *C. limon* revealed that genes associated with the accumulation of the primary metabolite citric acid are closely linked to DNA methylation levels. These genes included MYB transcription factors (ClPH1/4/5) and ANTHOCYANIN 1 (ClAN1) [71]. This may be related to the low levels of DNA methylation observed in the promoter region of *pleckstrin homologue 5* (*PH5*) [72]. It is suggested that there may be undiscovered links between DNA modifications and changes in primary and secondary metabolites during fruit ripening. Another key discovery is that DNA modifications transform the toxic, bitter compounds in *S. lycopersicum* fruit to nontoxic, nonbitter compounds during ripening. During fruit ripening, active DNA demethylation plays a key role in the biosynthesis of steroidal glycoalkaloids (SGAs). DNA demethylation, which is mediated by SlDML2, drives SGA detoxification during domestication [73]. Variation in DNA methylation may represent the genetic basis for this metabolic diversity in plants [74]. However, research on DNA modifications related to the biosynthesis of fruit secondary metabolites is still limited. The biosynthetic pathways of flavonoids remain poorly understood, and the regulatory roles of key structural genes remain unclear.

## Histone modifications

Histones are essential components of chromosomes. In eukaryotes, the highly conserved core histones H2A, H2B, H3, and H4 form the stable octameric core structure of nucleosomes, with H1 family members serving as linker histones [75]. Due to their complex structure, histones play a vital role in epigenetic regulation and gene expression control. The diverse array of modification types, sites, and variants collectively form the unique ‘histone code’ [76]. In addition to the classic methylation modification, numerous histone post-translational modifications (HPTMs), such as acetylation and ubiquitination, can also occur on the N-terminal ‘tail’ [13]. Currently, extensive research on histone modifications in horticultural plants has revealed that these modifications participate in crucial biological processes including growth [77], reproduction [78], biotic stress [79], and abiotic stress [80]. Here, we summarize the key regulatory networks governing histone modifications during fruit ripening and elucidate the relationships among these modifications (Fig. 2).

**Figure 2:**
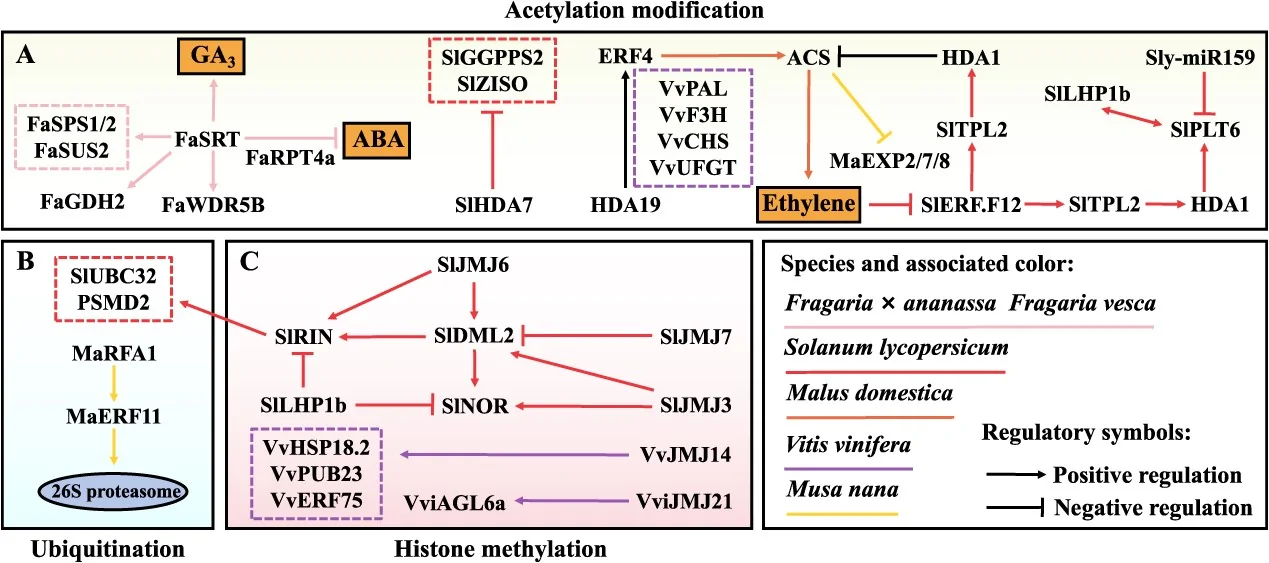
Regulation of histones and corresponding post-translational modifications during fruit ripening. The modifications include (A) acetylation, (B) ubiquitination, and (C) histone methylation. Fruits include tomato (*S. lycopersicum*), strawberry (*Fragaria* × *ananassa* and *F. vesca*), grape (*V. vinifera*), apple (*Ma. domestica*), and banana (*Mu. nana*). (A) The ‘HDA-ERF’ module influences climacteric fruit ripening and histone acetylation via regulation of ethylene signaling. The histone modification–related enzyme SRT influences nonclimacteric fruit ripening via participation in ABA hormone signaling pathways. (B) E3 ubiquitin ligase regulates the 26S proteasome pathway and undergoes ubiquitination modification during fruit ripening. (C) JMJ is a key enzyme influencing histone methylation during fruit ripening and is directly linked to active DNA demethylation. Black lines indicate regulatory pathways shared across multiple species. Positive regulation is indicated by arrows, while negative regulation is represented by T-shaped symbols.

JMJ is a key class of enzymes that influence histone methylation during fruit ripening. Histone methylation is a crucial form of epigenetic regulation. It primarily functions by regulating gene transcription, chromatin structure, and genomic stability [13]. Similar to the mechanism of DNA methylation, histone methylation is maintained at a certain level through the action of methyltransferases and demethylases [81–83]. Methylation modifications on histones H3K4, H3K36, and H3K79 are enriched in euchromatin, whereas those on H3K9, H3K27, and H4K20 typically accumulate in heterochromatin or facultative heterochromatin [84]. The ripening process of fruits is primarily regulated by the histone demethylase JMJ, which mainly affects two types of modifications: H3K4me3 and Histone H3 at lysine 27 trimethylation (H3K27me3). This histone methylation mechanism has been systematically studied in *S. lycopersicum* fruits and is associated with the RdDM pathway. Specifically, SlJMJ6 and SlJMJ7 can negatively regulate H3K27me3 [85] and H3K4me3 [60], respectively. This leads to histone demethylation in fruits and subsequently promotes ripening. This modification is strongly correlated with the DNA demethylase SlDML2 and participates in the regulation of SlRIN and SlNOR [61]. During this process, SlJMJ3 exhibits a positive correlation with SlDML2 and SlNOR [86]; heterochromatin protein 1b (SlHP1b) shows a negative correlation with SlDML2 and SlRIN [35], both mediated through H3K4me3 modification. In addition, loss of SlJMJ10 function resulted in smaller fruit, while its overexpression significantly increased fruit size by promoting epidermal cell expansion [87]. This role of JMJ enzymes in histone methylation has also been observed in *V. vinifera*, where they participate in fruit ripening. For example, VviAGL6a directly activates *VviJMJ21* expression by binding to its promoter, thereby promoting early ripening [88]. VvJMJ14 influences the transcriptional expression of *HEAT SHOCK PROTEIN* (*VvHSP18.2*), *PLANT U-BOX* (*VvPUB23*), and *ETHYLENE RESPONSE FACTOR* (*VvERF75*) by regulating the levels of H3K4me3 modification, thereby regulating fruit ripening [89]. However, studies on histone methylation modifications during fruit ripening in other species are rare.

Hormone signaling and histone deacetylases play crucial regulatory roles during fruit ripening. Histone acetylation on nucleosomes represents one of the earliest HPTMs studied in epigenetic regulation and is closely associated with gene activation [90]. Acetylation occurs on conserved lysine residues at the amino-terminal tail of core histones, with all four core histones capable of acetylation *in vivo* or *in vitro*. Generally, H3 and H4 are more readily acetylated than H2A and H2B, and each histone acetyltransferase modifies distinct lysine residues [91]. Histone deacetylase (HDA1) is a key enzyme for histone acetylation during the ripening of climacteric fruits. This pathway is primarily influenced by ethylene signalling, which negatively regulates SlERF.F12, a transcription factor containing the Ethylene-responsive element-binding factor-associated Amphiphilic Repression (EAR) motif. It suppresses the transcription of ripening-related genes by recruiting the corepressors TOPLESS 2 (TPL2) and SlHDA1 [92]. The SlPLT6 transcription factor contains the EAR motif and recruits SlHDA1 via its C-terminal domain. It also interacts with LIKE HETEROCHROMATIN PROTEIN 1b (SlLHP1b), providing a link to this maturation-inhibiting mechanism [93]. Additionally, MaERF11 recruits HDA1 during fruit ripening to suppress expression of acetyl-CoA synthetase (ACS) family [35] and directly binds to the promoters of the ripening-associated EXPORTIN family (*MaEXP2*/*7*/*8*) to reduce acetylation levels of histone H3 and H4 in *Musa nana* [94]. Response pathways regulating ethylene signaling via the ‘HDA-ERF’ module have also been found in *Ma. domestica* [95] and *V. vinifera* [96], influencing fruit ripening and anthocyanin biosynthesis. SIRTUINS (SRTs) may represent a key group of enzymes that influence histone modifications during nonclimacteric fruit ripening, which is primarily regulated by ABA signalling [97]. Among these, FaSRT1–2 actively promotes vegetative growth and fruit ripening in strawberry. This is achieved by regulating ABA and gibberellin (GA) signaling pathways [98]. FaSRT2–1 primarily influences the expression of genes such as *solanesyl diphosphate synthase* (*SPS*), *sucrose synthase* (*SUS*), and *human WDR5 homolog b* (*WDR5B*) by participating in energy metabolism [99]. However, our understanding of this histone deacetylase remains limited, as research on it has only recently begun and has primarily been conducted in *Fragaria* × *ananassa*. Does the SRT family perform the same function in other nonclimacteric fruits? What are the specific regulatory relationships between the SRT family and the hormones (ABA and GA)? Further research is required to answer these questions.

Protein ubiquitination occurs via the ubiquitin–proteasome pathway (UPP), where 76-amino-acid-long ubiquitin proteins are linked to lysine residues through amide bonds [100]. Histone ubiquitination primarily occurs on H2A and H2B. Histone H2A ubiquitination mainly targets lysine 119, primarily catalyzed by RING domain–containing E3 ubiquitin ligases [101]. This is a primary type of histone ubiquitination modification that affects fruit ripening. Research on ubiquitination during fruit ripening is currently limited, with only a few studies reported in *Mu. nana* and *S. lycopersicum*. Among these, the E3 ubiquitin ligase MaRFA1 accumulates and anchors MaERF11 during fruit ripening, causing a decrease in MaERF11 protein abundance and regulating the 26S proteasome pathway [102]. In addition, RIN transcription factors can directly bind to the promoters of *ubiquitin-conjugating enzyme* (*SlUBC32*) and 26S proteasome regulatory subunit (PSMD2); loss of RIN function affects protein ubiquitination within nuclei and consequently participates in the regulation of fruit ripening in *S. lycopersicum* [103]. This suggests that there might be significant regulatory crosstalk between histone methylation and ubiquitination during fruit ripening. However, research on the important links between these different histone modifications remains limited and has been conducted in only a few fruit species. Elucidating this key regulatory pathway may provide new insights into the epigenetic modifications involved in fruit ripening.

Histone modifications play a role in the synthesis and accumulation of various secondary metabolites during fruit ripening, including pigments, flavors, and aromatic compounds. This constitutes a complex epigenetic regulatory process. Firstly, histone modifications influence fruit color changes by regulating the dynamic balance of secondary metabolites, such as anthocyanins, carotenoids, and chlorophyll. In the synergistic regulation of DNA and histone methylation, low methylation levels induce increased expression of genes involved in photosynthesis, flavonoid biosynthesis, and hormone signaling pathways in *S. lycopersicum* and *F. vesca* fruits [104]. During the ubiquitination process, stearoyl-ACP desaturase 8 (SlSAD8) interacts with E3 ligase (SlSP1) within the chloroplast-associated protein degradation pathway in chloroplasts. This disrupts the normal function of the pathway and inhibits the transition of chloroplasts to chromoplasts [105]. During the process of acetylation modification, DREB and EAR motif protein (SlDEAR1) recruited Topless 2 (SlTPL2)-HDA1/HDA3 complex via its C-terminal EAR motif. This reduces the levels of histone acetylation in the SHOOT GRAVITROPISM 1 (SlSGR1) promoter region and regulates chlorophyll metabolism in fruit [106]. This ‘TPL-HDA1/HDA3 complex’ module also reduced histone acetylation levels in lycopene biosynthesis genes [107]. Similar findings were observed in *Ma. domestica* fruit, where transcription factors containing the EAR motif suppressed carotenoid biosynthesis via a cascade regulated by TPL-mediated histone deacetylation [108]. Unlike the regulatory mechanisms of other secondary metabolites, the expression of genes involved in fruit anthocyanin synthesis is primarily influenced by the inhibitory effect of histone deacetylases binding to ERF transcription factors on GCC-box [109], particularly the MYB transcription factor family [68]. Secondly, histone modifications might influence the release of aromatic compounds in fruits. A classic example is the interaction identified between PpMADS2 and E3 ubiquitin ligase CONSTITUTIVE PHOTOMORPHOGENIC 1 (PpCOP1) in *P. persica* L. Batsch. This module regulated linalool biosynthesis by activating the expression of PpTPS1, a terpene synthase [110]. This phenomenon of aroma substance release may also be regulated during DNA and histone methylation processes [104]. In addition, SlDML2-mediated DNA demethylation regulated the SGA detoxification process in *S. lycopersicum* fruit, transforming it into a nontoxic and nonbitter state, which was a significant advancement in recent studies of fruit epigenetic modification [73]. This process is primarily regulated by the GLYCOALKALOID METABOLISM (SlGAME) genes. The acetylation of SGAs in cultivated and wild tomatoes is catalyzed by the BAHD-type acyltransferase GAME36, which is involved in the regulation of α-tomatine biosynthesis and degradation [111]. This suggests that the detoxification pathway of SGA may be jointly regulated by multiple epigenetic modifications. Although detailed studies have been conducted on acetylation modifications involved in the production of secondary metabolites in fruits, some pathways remain unidentified. For example, the accumulation of the primary metabolite citric acid in citrus fruits correlated with low levels of DNA methylation of *PH5* [72]. Meanwhile, histone acetyltransferases (CitHAG11/28) show a predominantly negative correlation with citric acid content [112]. Is there a regulatory pathway linking these two processes? Overexpression of lysine deacetylase during polyploidization shifts metabolic flux from primary to secondary metabolism, with acetylation reprogramming playing a crucial role in this process [113]. Does this reprogramming affect the conversion of citrate into other secondary metabolites? These changes in epigenetic modifications may also suggest the existence of significant connections. Such variations may also indicate important connections in this process.

## RNA modifications

RNA modification refers to the various chemical modifications that occur on RNA and are widely found in the four nucleotides A, U, C, and G. Among these, methylation modifications on RNA nucleosides constitute one of the primary forms of RNA modification, including *N^1^*-methyladenosine (m^1^A), 3-methylcytosine (m^3^C), 5-methylcytosine (m^5^C), *N^6^*-methyladenine (m^6^A), and *N^7^*-methylguanine (m^7^G), among others [114]. Studies have shown that RNA methylation modifications are dynamically reversible just like DNA methylation modifications, and elevating RNA modifications from a fine-tuning mechanism to a new regulatory level known as the epitranscriptome. In fact, it does not fall within the scope of classical epigenetics. Further research into the characteristics and regulatory mechanisms of RNA modifications will enhance our understanding of the biology of RNA modifications from the perspective of epitranscriptomics. During fruit ripening, research on RNA methylation modifications with m^6^A at its core has gradually advanced, and whole-genome RNA methylation maps for m^1^A and m^5^C have been constructed. Additionally, some attention has been directed toward RNA acetylation modifications involving *N^4^*-acetylcytidine (ac^4^C) (Fig. 3).

**Figure 3:**
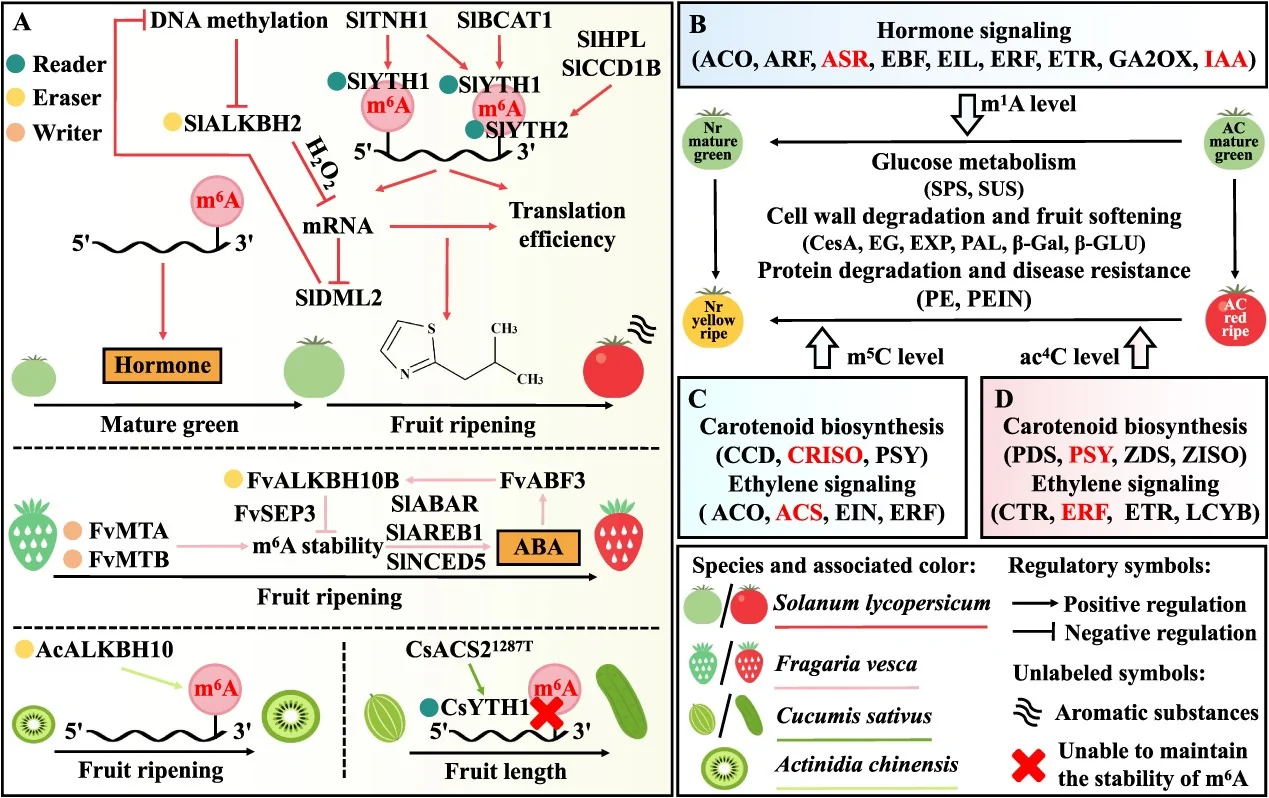
RNA modifications during fruit ripening. Modifications include (A) m^6^A, (B) m^1^A, (C) m^5^C, and (D) ac^4^C. Fruits include tomato (*S. lycopersicum*), strawberry (*F. vesca*), kiwifruit (*A. chinensis*), and cucumber (*C. sativus*). (A) ‘Eraser’ enzymes primarily regulate fruit enlargement and ripening; ‘reader’ enzymes mainly participate in the synthesis of aromatic volatiles and fruit length regulation. (B) The m^1^A methylation levels decrease during postveraison in *S. lycopersicum*, while multiple genes associated with ethylene signaling are activated. (C) Elevated m^5^C levels activate mRNAs involved in cell wall biosynthesis and secondary metabolite accumulation during the ripening of *S. lycopersicum*. (D) The ac^4^C modification during postveraison significantly alters the expression of genes related to carotenoid biosynthesis, ethylene signaling, and cell wall construction in *S. lycopersicum* fruits. Positive regulation is indicated by arrows, while negative regulation is represented by T-shaped symbols.

m^6^A modification influences fruit ripening, aroma compound release, and biological trait changes. This is the most prevalent form of methylation in eukaryotic RNA, occurring at the sixth nitrogen atom of the adenine base [115]. Chemically, this modification is highly stable, insensitive to chemical reagents, and does not affect nucleotide base pairing. With the advancement and application of high-throughput sequencing methods, three classes of methyltransferases involved in plant m^6^A modification have been progressively identified: ‘writers’, ‘erasers’, and ‘readers’ [116]. ‘Writers’ primarily regulate m^6^A deposition, including mRNA adenosine methylase (MTA), METHYLTRANSFERASE B (MTB), FKBP12-interacting protein 37 (FIP37), VIRILIZER (VIR), and HAKAI [117]. ‘Erasers’ represent a crucial class of demethylases, primarily mediated by the ALKB HOMOLOG (ALKBH) family in plants [118]. ‘Readers’ possess a YTH conserved domain, with *A. thaliana* harboring 13 members, which is significantly more than the five found in humans [119]. Firstly, these three classes of methyltransferases involved in m^6^A modification play a critical and synergistic role during fruit ripening [120–123]. For example, in *S. lycopersicum*, ‘erasers’ proteins are primarily associated with fruit enlargement and ripening; ‘readers’ proteins are primarily associated with the synthesis of aromatic volatiles. From immature green stage to green-ripe mature stage, overall m^6^A levels increase during fruit enlargement in *S. lycopersicum*, with thousands of protein-coding genes undergoing m^6^A modification in 3′ untranslated regions (UTRs) [124]. This is primarily regulated by SlALKBH2, which is sensitive to H_2_O_2_-mediated oxidative modifications. SlALKBH2 promotes protein stability by forming homodimers via intermolecular disulfide bonds [125]. Mutations in SlALKBH2 regulate the DNA demethylase SlDML2 and delay fruit ripening [126]. In addition, ‘reader’ protein controls the release of aroma compounds in *S. lycopersicum* fruit. Hydroperoxide lyase (SlHPL) and carotenoid cleavage dioxygenase 1B (SlCCD1B), which are associated with the synthesis of volatile compounds, accumulate in SlYTH2-induced aggregates, and negatively regulate fruit aroma [127]. SlYTH1 exerts an opposite effect to SlYTH2 in regulating the release of aromatic compounds. Knockout of SlYTH1 specifically reduces the content of flavor-related volatiles in fruits without affecting ripening time, ethylene production, or fruit firmness [128]. Obtaining fruits with intense aroma without compromising yield and quality may represent a key target for future molecular breeding efforts. Significant m^6^A modifications in *Actinidia chinensis* also occur at stop codons and in 3′UTR, with m^6^A levels gradually decreasing during fruit ripening. Among these, AcALKBH10 plays a crucial regulatory role in soluble sugar accumulation [129]. Furthermore, a recent study revealed a dominant interaction between YTH1 and ACS2. In wild cucumber (*Cucumis sativus* var. *hardwickii*), *ACS2^1287C^* mutation triggered the addition of an m^6^A modification to a neighboring adenosine residue, which was recognized by YTH1. The equilibrium of RNA folding was shifted towards the weakest structural conformation, which resulted in shorter fruit. In contrast, in cultivated cucumber (*C. sativus*), *ACS2^1287T^* mutation disrupted m^6^A methylation, formed a more compact RNA conformation, and resulted in elongated fruit [130]. This discovery provides crucial genetic evidence that epitranscriptomic regulation shapes biological traits. Secondly, m^6^A modification primarily affects mRNA stability during fruit ripening. In *S. lycopersicum*, SlALKBH2 primarily inhibits fruit ripening by reducing the mRNA abundance of the DNA demethylase SlDML2 [126]. During the onset of fruit ripening in *F. vesca*, m^6^A modifications in coding sequence (CDS) regions tend to stabilize mRNA, whereas m^6^A at stop codons and in 3′UTR shows a negative correlation with the abundance of associated mRNA. FvMTA and FvMTB are indispensable in this process, as MTA-mediated m^6^A modification enhances the mRNA stability of NINE-CIS-EPOXYCAROTENOID DIOXYGENASE 5 (FvNCED5) and ABSCISIC ACID RESPONSIVE ELEMENTS–BINDING PROTEIN (FvAREB1) [131]. Finally, m^6^A modification is involved in the hormonal signaling pathways that regulate fruit ripening. Studies in *S. lycopersicum* have shown that overall m^6^A levels increase during fruit enlargement regulated by SlALKBH2, and genes associated with the ethylene signaling pathway are activated and expressed [124, 125]. In typical nonclimacteric fruits *F. vesca*, ‘writer’ and ‘eraser’ proteins participate in ABA signalling pathway through positive and negative regulation [131]. FvALKBH10B is also activated by ABA and positively regulates the stability of SEPALLATA3 (FvSEP3) mRNA via m^6^A demethylation. Mutations in FvALKBH10B cause delayed fruit ripening and induce hypermethylation of m^6^A in 1859 genes [132]. Although a series of studies have investigated the regulatory mechanisms of m^6^A modification in fruits, research on its role in key secondary metabolite biosynthesis and color-related substance changes remains insufficient, with limited fruit types studied. This is primarily constrained by the difficulty in obtaining perennial woody fruit tree materials and the lack of stable genetic transformation techniques.

RNA modifications participate in cell wall construction and hormone signaling pathways during fruit ripening. In addition to m^6^A modifications, RNA methylation modifications (including m^1^A and m^3^C), as well as RNA acetylation modifications (such as ac^4^C), have been identified during fruit ripening. m^1^A is an RNA methylation modification occurring on the first nitrogen atom of the adenine base. This modification is primarily distributed in the first exon of coding sequences. Unlike m^6^A, m^1^A-modified sequences lack distinct characteristics, exhibit Guanine-Cytosine (GC) base enrichment around modification sites, and have lower minimum free energy [133]. m^1^A modification is widely present in *S. lycopersicum* mRNAs and can also be detected in long noncoding RNAs (lncRNAs) and circular RNAs (circRNAs), which are primarily enriched in start codons and coding regions. From the green-ripe stage to 6 days postveraison, the overall m^1^A methylation level in fruit decreases, a process accompanied by significant differential expression of genes associated with hormone signaling pathways [134]. m^5^C, a highly dynamic methylation modification on mRNA, shows significant enrichment at translation initiation sites in human [135] and mouse [136] cells, but has not been detected in *A. thaliana* [137]. Compared to m^6^A, research on m^5^C is still in its infancy, and its specific regulatory mechanisms remain to be elucidated. mRNAs with elevated levels of m^5^C participate in responses to biotic/abiotic stress and cell wall metabolism during fruit ripening in Alisa Craig (AC). In the Never Ripe (Nr) mutant fruit, the expression of genes associated with carotenoid biosynthesis and ethylene signaling is primarily observed, alongside the identification of lncRNA and circRNA targets bearing distinct m^5^C sites [138]. ac^4^C is a highly conserved RNA acetylation modification widely present in transfer RNA (tRNA) and ribosomal RNA (rRNA), and has also been progressively identified in mRNA [139]. The presence of ac^4^C acetylation has also been confirmed in plant mRNAs, such as those in *A. thaliana* and *O. sativa* [140]. From the green-ripe stage to 6 days after color break, acetylation levels of AC and Nr showed decreasing and increasing trends, respectively, with significant differential expression of genes related to carotenoid biosynthesis, ethylene signaling, and cell wall construction. Among these, the ac^4^C levels of key genes SUS, aconitase (ACO), ethylene response (ETR), and cellulose synthase (CesA) were down-regulated, while the ac^4^C level of β-galactosidase (β-Gal) was up-regulated. Correspondingly, lncRNA also exerted positive regulatory effects on target gene expression [141]. Since these RNA modifications have all been discovered and studied in recent years, research on fruit ripening has primarily focused on mapping genome-wide RNA modification profiles. These emerging RNA modifications may resolve existing biological questions and reveal novel regulatory mechanisms in the future.

## Applications of epigenetic modification inhibitors

Unlike DNA variations such as mutations, epigenetic modifications are reversible and can be modulated by approaches such as exogenous chemical treatment, a strategy known as ‘epigenetic therapy’. Currently, multiple epigenetic modification inhibitors have been approved for clinical trials and disease treatment [142]. During fruit ripening, DNA methylation inhibitors (5-aza-2′-deoxycytidine, 5-aza-2dC; 5-azacytidine, 5-azaC), histone modification inhibitors (Trichostatin A, TSA; Sodium butyrate, NaB; Sirtinol; 5′-deoxy-5′-methylthioadenosine, MTA), and RNA modification inhibitors (3-deazaneplanocin A, DA; meclofenamic acid, MA) have been applied (Table 1).

**Table 1:** The effects of epigenetic modification inhibitors in fruits.

**Species**	**Names**	**Types**	**Functions**	**Regulatory relationship**	**References**
*Ma. domestica*	5-aza-2dC	DNA methylation inhibitor	Increased anthocyanin content in fruit	Negative correlation between DNA methylation and gene expression in the flavonoid pathway	[68]
*S. lycopersicum*	5-azaC	DNA methylation inhibitor	Promoted early fruit ripening; reduced carotenoids; increased the accumulation of ascorbic acid in fruit; reduced susceptibility to gray mold	Decreased DNA methylation levels; RIN transcription factors bind to the promoters of genes associated with fruit ripening; SlGalUR5 involved in ascorbic acid accumulation; increased expression of *SlDCL2*	[143–145]
*Vitis labruscana* (*V. labrusca* × *V. vinifera*)	5-azaC	DNA methylation inhibitor	Promoted fruit ripening	Decreased DNA methylation levels; Genes associated with fruit ripening, photosynthesis, protein phosphorylation, and heat stress are regulated	[146, 147]
*C. sinensis* Osbeck	5-azaC	DNA methylation inhibitor	Inhibited fruit peel yellowing	Decreased DNA methylation levels	[51]
*P. persica*	5-azaC	DNA methylation inhibitor	Increased the anthocyanin content of fruit pulp	Decreased DNA methylation levels; genes regulating anthocyanin biosynthesis are suppressed	[70]
*Fragaria* × *ananassa*	5-azaC	DNA methylation inhibitor	Reduced carotenoid and anthocyanin contents; inhibited fruit ripening	Decreased DNA methylation levels	[104]
*P. bretschneideri*	5-azaC	DNA methylation inhibitor	Increased levels of abscisic acid, β-carotene, and lutein in fruit pulp	Decreased DNA methylation levels; increased expression of ABA biosynthesis genes	[65]
*C. sinensis* (Linn.) Osbeck	5-azaC	DNA methylation inhibitor	Increased the anthocyanin content of fruit pulp	Decreased DNA methylation levels; reduced levels of DNA methylation in the promoter regions of genes associated with anthocyanin synthesis and accumulation	[69]
*V. labruscana* (*V. labrusca* × *V. vinifera*)	5-azaC TSA	DNA methylation inhibitor Histone deacetylase inhibitor	TSA delayed the depletion of organic acids in postharvest grapes, whereas 5-azaC had the opposite effect	*VvHDA15* affected fruit quality after harvest through acetylation modification	[148]
*V. vinifera*	TSA	Histone deacetylase inhibitor	Increased the anthocyanin content of fruit pulp	VvERF4 interacted with VvHDAC19, and together downregulated the expression of target genes involved in anthocyanin biosynthesis	[96]
*Citrus clementina* hort. (*Citrus paradisi* × *Citrus reticulata*)	NaB Sirtinol	Histone deacetylase inhibitor	Increased the anthocyanin content	The inhibitors did not inhibit the expression level of *CgSRT2* and *CgHDA8*	[113]
*V. labruscana* (*V. labrusca* × *V. vinifera*)	Sirtinol	Histone deacetylase inhibitor	Increased the anthocyanin content of fruit peel	Post-translational modifications occurred in anthocyanin biosynthesis–related genes	[149]
*V. vinifera*	MTA	Histone methyltransferase inhibitor	Promoted early fruit ripening	VviAGL6a directly bound to *VviJMJ21* promoter	[88]
*S. lycopersicum*	DA MA	m^6^A writer inhibitor m^6^A eraser inhibitor	Inhibited fruit enlargement; DA promoted fruit ripening; MA inhibited fruit ripening	The m^6^A modification genes involved in fruit enlargement were regulated by DA or MA	[124]

5-azaC is a base analog that reduces genomic DNA methylation levels by inhibiting methyltransferase activity. Low concentrations of 5-azaC effectively reduce DNA methylation levels by decreasing intracellular DNA methyltransferase activity, while high concentrations participate in RNA or DNA synthesis and exert cytotoxic effects [142]. Exogenous 5-azaC treatment significantly reduces genome-wide DNA methylation during the ripening process of fruits. Application methods for this substance include surface coating [150], spray application on fruit clusters [148], and fruit injection [104]. Exogenous application of 5-azaC to climacteric fruits promotes ripening, increases anthocyanin content, and enhances the accumulation of substances related to disease resistance [143–145]. Exogenous application of 5-azaC to nonclimacteric fruits induces premature ripening in *V. labruscana*, with significant expression of genes associated with fruit softening [146, 147]. This contrasts with the opposite effect observed in *Fragaria* × *ananassa* fruits [104]. Furthermore, this DNA methylation inhibitor affected secondary metabolite accumulation [51, 69] in citrus. In addition, 5-aza-2′-deoxycytidine (5-aza-2dC) has been found to promote anthocyanin accumulation in the peel of *Ma. domestica* [68]. It is a 2′-deoxycytidine analogue that exhibits lower toxicity than 5-azaC [142]. TSA, a histone deacetylase inhibitor, is used to regulate gene expression at the transcriptional level. It induces a series of biological effects, including chromatin remodeling through protein hyperacetylation, induction of cell differentiation, and modulation of transcription factor activation/inhibition [151]. Exogenous application of TSA in *V. labruscana* delayed the depletion of organic acids and soluble sugars in fruit, whereas 5-azaC treatment produced the opposite effect; both inhibitors suppressed linalool release, reducing the musky aroma of fruit [148]. Furthermore, exogenous TSA application in *V. vinifera* affected anthocyanin accumulation [96], a finding mirrored in fruits treated with other histone deacetylase inhibitors (NaB, Sirtinol) [113, 149]. In contrast, the histone methyltransferase inhibitor MTA primarily influenced fruit ripening processes [88]. An overall increase in m^6^A levels, primarily regulated by m^6^A modifications occurring in the 3′UTR through the action of ‘writer’ and ‘eraser’ proteins, is frequently observed during fruit enlargement in *S. lycopersicum*. Injecting fruit with inhibitors of ‘writers’ (DA) and ‘erasers’ (MA) of m^6^A inhibits fruit enlargement, while DA simultaneously promotes fruit ripening [124]. This indicates that fruit ripening may be impacted by various epigenetic alterations and that these effects vary in aspects such as physical development, color changes, and the emission of aromatic substances. In addition, some of the epigenetic modifiers currently in use exhibit a certain degree of toxicity. Among these, 5-azaC, which is widely used in plant research, is particularly toxic and carcinogenic [152]. Are they truly suitable for regulating fruit ripening via injection or soaking? On the one hand, this could raise consumer concerns about food safety. On the other hand, these epigenetic modification inhibitors are all quite expensive, which also significantly increases agricultural production costs.

**Figure 4:**
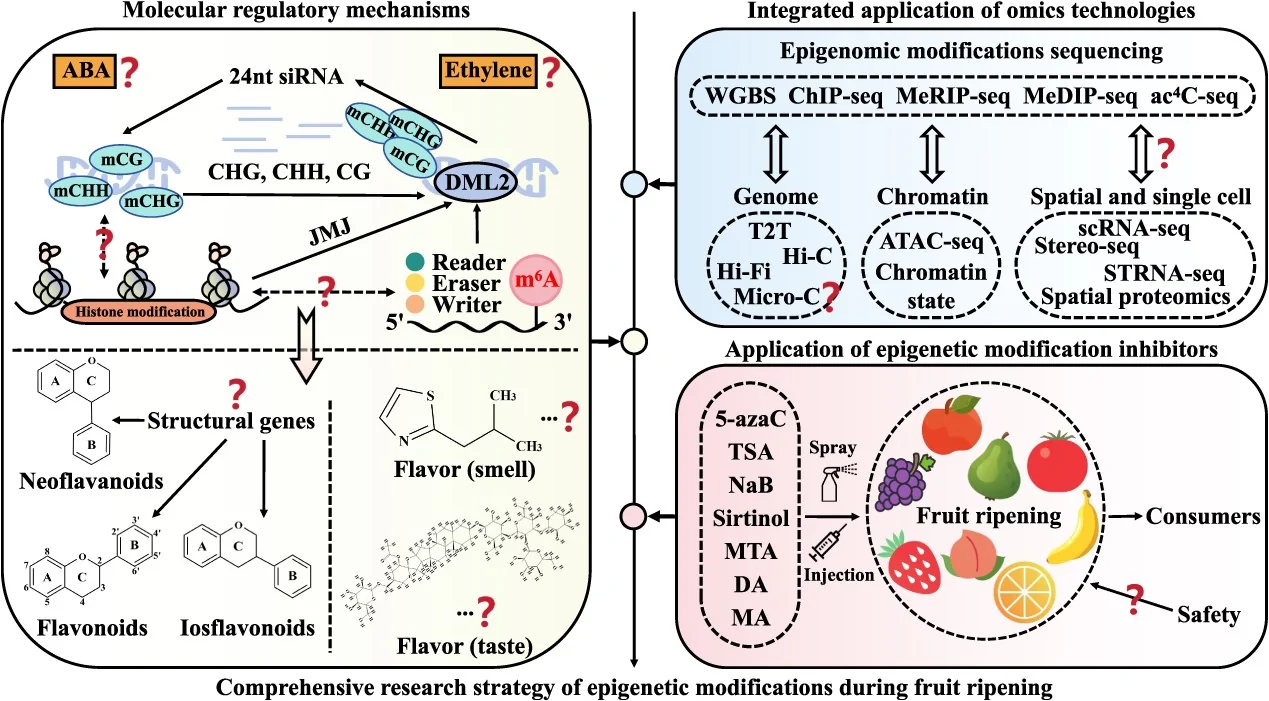
A comprehensive strategy model for research on epigenetic modifications during fruit ripening. It comprises three components: the integrated application of omics technologies, the exploration of molecular regulatory mechanisms, and the application of epigenetic modification inhibitors. Firstly, combine epigenetic sequencing technologies with novel sequencing techniques at the genomic, chromatin, spatial, and single-cell levels to map the epigenetic modifications influencing fruit ripening through multiomics integration analysis. Secondly, based on these multiomics epigenetic maps, focus on deciphering the unknown regulatory relationships among DNA, histone, and RNA modifications during fruit ripening. Clarify how epigenetic modifications influence the ripening process of both climacteric and nonclimacteric fruits via ethylene and ABA signaling pathways. Further investigate the effects of epigenetic modifications on changes in fruit secondary metabolism, as well as previously undiscovered regulatory relationships involving structural genes. Finally, evaluate the impacts of exogenous epigenetic modification inhibitors on food safety and cultivation costs, and screen for harmless regulatory systems for fruit ripening that are suitable for agricultural production. The areas marked with ‘?’ represent currently unresolved issues in the study of epigenetic modifications during fruit ripening.

## Application of epigenetic modification sequencing technology

With the continuous advancement of epigenetic research, numerous sequencing technologies for epigenetic modifications have been developed. Based on varying detection requirements and sample types, epigenetic modification sequencing has progressively expanded from single-gene loci to multigene loci and ultimately to genome-wide loci. Using fruits as sequencing materials, comprehensive epigenetic modification sequencing has been performed across DNA, histone, and RNA levels, significantly advancing research into fruit quality and ripening processes (Table 2).

**Table 2:** Application of sequencing technologies associated with epigenetic modifications in fruits.

**Species**	**Sequencing technologies**	**Analysis types**	**Analysis scope**	**References**
*S. lycopersicum*	WGBS ChIP-seq	DNA methylation	Whole-genome, H3K4me3, H3K27me3, H3K27ac, H3K9/K14ac	[59, 73, 74]
*Fragaria × ananassa*	WGBS	DNA methylation	Whole-genome	[53]
*C. sinensis* Osbeck	WGBS	DNA methylation	Whole-genome	[51]
*Ma. domestica*	WGBS	DNA methylation	Whole-genome	[66, 68]
*Citrus limon*	WGBS	DNA methylation	Whole-genome	[71]
*Pyrus pyrifolia*	WGBS	DNA methylation	CG and CHG methylation	[64]
*P. bretschneideri*	WGBS	DNA methylation	Whole-genome	[65]
*V. vinifera*	WGBS	DNA methylation	Whole-genome	[50, 53]
*P. persica*	WGBS	DNA methylation	Whole-genome	[58]
*C. sinensis* L. Osbeck	WGBS	DNA methylation	Whole-genome	[69]
(*V. bryoniifolia × V. vinifera*) *× V. vinifera*	WGBS	DNA methylation	Whole-genome	[67]
*V. labrusca* × *V. vinifera*	WGBS	DNA methylation	CHH hypermethylation	[52]
*S. lycopersicum*	ChIP-seq	Histone modification	H3K4me3, H3K9Ac, and H3K27Ac	[60, 85, 92]
*V. vinifera*	ChIP-seq	Histone modification	H3K4me3	[89]
*Ma. domestica*	ChIP-seq	Histone modification	H3K9ac	[95]
*P. pyrifolia* × *Pyrus communis*	ChIP-seq	Histone modification	H3	[109]
*F. vesca*	ChIP-seq Chromatin state analysis	Histone modification	H3K9/K14ac, H3K27ac, H3K4me3, and H3K36me3	[97]
*Mu. acuminata*	DNase-seq ChIP-seq	Histone modification	H3K4me3, H3K9ac, H3K27ac, and H3K27me3	[153]
*S. lycopersicum*	MeRIP-seq MeDIP-seq ac^4^C-seq	RNA modifications	m^6^A modifications m^1^A modifications RNA m^5^C methylation ac^4^C methylation	[124–126, 128, 134, 138, 141, 154]
*F. vesca*	MeRIP-seq	RNA modifications	m^6^A modifications	[129, 130]
*Actinidia chinensis*	MeRIP-seq	RNA modifications	m^6^A modifications	[131, 155]
*Cucumis sativus*	MeRIP-seq	RNA modifications	m^6^A modifications	[132]

Whole-genome bisulfite sequencing (WGBS) technology combines bisulfite treatment with high-throughput sequencing to detect individual cytosine methylation levels across the entire genome. It is currently the most widely applied DNA methylation sequencing technique in plants [156]. This method enables high-coverage screening of unknown DNA methylation regions in samples, including low-CpG-density areas. Extensive WGBS analyses have been conducted on fruits, covering major cultivated fruit species worldwide. However, fruit ripening is often accompanied by tissue specificity and varying growth rates. Currently, the application of sequencing technologies for DNA methylation analysis largely relies on the whole-genome level rather than specific regions, which limits the utility of the resulting epigenetic modification profiles and may lead to inconsistencies with transcriptomic data. Additionally, a combined analysis of WGBS and Chromatin Immunoprecipitation Sequencing (ChIP-seq) has been conducted in *S. lycopersicum*, enabling systematic and in-depth data mining of regions modified by H3K4me3, H3K27me3, Histone H3 lysine 27 acetylation (H3K27ac), and histone H3 lysine 9 acetylation (H3K9ac) [73]. ChIP-seq is a research method that combines ChIP technology with next-generation sequencing to reveal DNA–protein interactions. It has been widely applied in studies of RNA polymerase [157], histone modifications [158], and the mechanisms underlying transcription factor function and regulation [59], playing a significant role in investigating gene expression in eukaryotes. In *S. lycopersicum* [60, 85, 92], *Ma. domestica* [95], and *P. pyrifolia* × *P. communis* [109] fruits, ChIP-seq technology has been employed to analyze specific histone modification regions, particularly those involving histone H3. Furthermore, this sequencing technology has begun to be integrated with other omics techniques for combined omics analysis, such as Chromatin State Analysis [97] and DNase I Hypersensitive Sites Sequencing (DNase-seq) [153]. The combination of multiomics approaches with genome-wide or site-specific analyses has largely overcome the limitations imposed by the unique characteristics of the material or the scope of detection. However, this approach remains underutilized in studies of fruit ripening, with research primarily relying on ChIP-seq or ChIP-qPCR at specific sites. Methylated RNA immunoprecipitation sequencing (MeRIP-seq) is the earliest developed and most widely applied m^6^A sequencing technology. It utilizes specific antibodies to enrich RNA containing m^6^A, addressing the challenge that this modification does not affect nucleotide base pairing capacity and is insensitive to chemical reagents [115]. Currently, MeRIP-seq has become the primary technique for studying m^6^A modifications that influence fruit ripening processes and quality. In addition, techniques such as methylated DNA immunoprecipitation sequencing (MeDIP-seq) [134, 138] and *N^4^*-acetylcytidine sequencing (ac^4^C-seq) [141], which are used to study RNA methylation and acetylation, respectively, have recently been applied. These approaches may also emerge as important methods for addressing existing biological questions in the future. Overall, multitaxon and multiomics population studies have become the primary approach for elucidating plant evolution and molecular regulatory mechanisms in the postgenomic era. Integrating epigenomic sequencing with other omics technologies has emerged as a key strategy for deciphering fruit ripening processes and quality changes.

## Conclusion

Research on DNA, histone, and RNA modifications has significantly advanced our understanding of the processes and quality changes involved in fruit ripening, particularly with regard to elucidating the molecular regulatory networks. However, studies on epigenetic modifications during fruit ripening continue to present opportunities and challenges. A comprehensive strategy is therefore required to investigate these modifications (Fig. 4). Firstly, combine epigenetic modifications during fruit ripening with multiomics technologies, such as genomic sequencing represented by telomere-to-telomere (T2T), high-throughput/resolution chromosome conformation capture (Hi-C), High-Fidelity Sequencing (Hi-Fi), and Micrococcal Nuclease-based Chromosome Conformation Capture (Micro-C); chromatin conformation studies represented by Assay for Transposase-Accessible Chromatin with high-throughput sequencing (ATAC-seq) and chromatin state analysis; high-resolution histone sequencing studies represented by Cleavage Under Targets and Tagmentation (CUT&Tag) and Cleavage Under Targets and Release Using Nuclease (CUT&RUN); and single-cell and spatial omics studies represented by single-cell transcriptome (scRNA-seq), spatial enhanced resolution omics sequencing (Stereo-seq), spatial transcriptomics RNA sequencing (STRNA-seq), and spatial proteomics. The introduction of these technologies will significantly enhance our understanding of the deeper epigenetic modifications and their corresponding regulatory relationships during fruit ripening. Secondly, elucidate the profound relationships among DNA, histone, and RNA modifications. Future research should focus on interpreting the tentative findings and uncovering the unidentified regulatory roles identified in these preliminary investigations. For example, the deep synergies between epigenetic modifications and transcriptional activation, mRNA stability, or hormone signaling; how changes in DNA methylation affect chromatin accessibility; whether histone acetylation and methylation events precede or accompany DNA demethylation; and mechanisms by which epigenetic modifications influence the biosynthesis pathways of secondary metabolites by regulating key structural genes. New epigenetic editing tools (such as CRISPR-dCas9-based DNA demethylation or histone modification systems) may help address these critical biological questions. This approach can help improve fruit development processes and quality through the regulation of epigenetic modifications, and drive innovation in molecular breeding. Finally, it is crucial to clarify the advantages and disadvantages of applying epigenetic modification inhibitors, which are a ‘double-edged sword’. If applied externally via injection, immersion, or topical application, could toxic residues remain in the fruit? Are there safer, effective alternatives that can achieve these changes in epigenetic modification levels? Addressing these questions will significantly improve the feasibility of translating epigenetic modification research from the laboratory to practical agricultural production.

A comprehensive strategy integrating studies on multiple epigenetic modifications is crucial for addressing current limitations. This approach will clarify unknown molecular regulatory mechanisms while enabling the application of these novel technologies to regulate fruit ripening timing, improve postharvest preservation, and extend shelf life in horticultural fruit plants. By focusing on these understudied areas, we will further advance our understanding of fruit ripening processes and refine existing integrated regulatory strategies.

## Data Availability

All data are available in the tables and figures of the manuscript.

## References

[1] Gebara CH, Berthet E, Vandenabeele M. et al. Diets can be consistent with planetary limits and health targets at the individual level. Nat Food. 2025;6:466–77 10.1038/s43016-025-01133-y 40119219

[2] Cai H, Xuan J, Wang X. et al. The multiple benefits of Chinese dietary transformation. Nat Sustainability. 2025;8:606–18

[3] Wang X, Dou Z, Feng S. et al. Global food nutrients analysis reveals alarming gaps and daunting challenges. Nat Food. 2023;4:1007–17 10.1038/s43016-023-00851-5 37828076

[4] Passarelli S, Free CM, Shepon A. et al. Global estimation of dietary micronutrient inadequacies: a modelling analysis. Lancet Glob Health. 2024;12:e1590–9 10.1016/S2214-109X(24)00276-6 PMC11426101 39218000

[5] Xu X, Yan P, Chen W. et al. The global burden of disease attributable to suboptimal fruit and vegetable intake, 1990-2021: a systematic analysis of the global burden of disease study. BMC Med. 2025;23:456 10.1186/s12916-025-04275-9 PMC12326680 40764991

[6] Yang C, Ying S, Tang B. et al. The mechanistic insights into fruit ripening: integrating phytohormones, transcription factors, and epigenetic modification. J Genet Genomics. 2025;52:1475–89 10.1016/j.jgg.2025.06.001 40581252

[7] Chen T, Ji D, Zhang Z. et al. Advances and strategies for controlling the quality and safety of postharvest fruit. Engineering. 2021;7:1177–84

[8] Liu X, Bourvellec CL, Yu J. et al. Trends and challenges on fruit and vegetable processing: insights into sustainable, traceable, precise, healthy, intelligent, personalized and local innovative food products. Trends Food Sci Technol. 2022;125:12–25

[9] Huang W, Hu N, Xiao Z. et al. A molecular framework of ethylene-mediated fruit growth and ripening processes in tomato. Plant Cell. 2022;34:3280–300 10.1093/plcell/koac146 PMC9421474 35604102

[10] Zhu F, Wen W, Cheng Y. et al. The metabolic changes that effect fruit quality during tomato fruit ripening. Mol Hortic. 2022;2:2 10.1186/s43897-022-00024-1 PMC10515270 37789428

[11] Zhang RX, Liu Y, Xiao W. et al. Diverse actions of auxin on fruit development and ripening. Hortic Adv. 2025;3:29

[12] Wang W, Fan D, Hao Q. et al. Signal transduction in non-climacteric fruit ripening. Hortic Res. 2022;9:uhac190 10.1093/hr/uhac190 PMC9622361 36329721

[13] Wang W, Wang Y, Chen T. et al. Current insights into posttranscriptional regulation of fleshy fruit ripening. Plant Physiol. 2023;192:1785–98 10.1093/plphys/kiac483 PMC10315313 36250906

[14] Waddington CH . The epigenotype. Endeavour. 1942;1:18–20

[15] Allis CD, Jenuwein T, Reinberg D. et al. Epigenetics. Cold Spring Harbor, New York: Cold Spring Harbor Laboratory Press; 2007:

[16] Xue Y, Cao X, Chen X. et al. Epigenetics in the modern era of crop improvements. Sci China Life Sci. 2025;68:1570–609 10.1007/s11427-024-2784-3 39808224

[17] Wang S, Wang M, Ichino L. et al. MBD2 couples DNA methylation to transposable element silencing during male gametogenesis. Nat Plants. 2024;10:13–24 10.1038/s41477-023-01599-3 PMC10808059 38225352

[18] Hu H, Du J. Structure and mechanism of histone methylation dynamics in *Arabidopsis*. Curr Opin Plant Biol. 2022;67:102211 10.1016/j.pbi.2022.102211 35452951

[19] Zhong Z, Wang Y, Wang M. et al. Histone chaperone ASF1 mediates H3.3-H4 deposition in *Arabidopsis*. Nat Commun. 2022;13:6970 10.1038/s41467-022-34648-0 PMC9666630 36379930

[20] Meng X, Yu X, Wu Y. et al. Chromatin remodeling protein ZmCHB101 regulates nitrate-responsive gene expression in maize. Front Plant Sci. 2020;11:52 10.3389/fpls.2020.00052 PMC7031486 32117389

[21] Hoang NT, Toth K, Stacey G. The role of microRNAs in the legume-rhizobium nitrogen-fixing symbiosis. J Exp Bot. 2020;71:1668–80 10.1093/jxb/eraa018 32163588

[22] Cheng P, Bao S, Li C. et al. RNA *N^6^*-methyladenosine modification promotes auxin biosynthesis required for male meiosis in rice. Dev Cell. 2022;57:246–259.e4 10.1016/j.devcel.2021.12.014 35026163

[23] Zhu W, Hu B, Becker C. et al. Altered chromatin compaction and histone methylation drive non-additive gene expression in an interspecific *Arabidopsis* hybrid. Genome Biol. 2017;18:157 10.1186/s13059-017-1281-4 PMC5568265 28830561

[24] Park K, Kim MY, Vickers M. et al. DNA demethylation is initiated in the central cells of *Arabidopsis* and rice. Proc Natl Acad Sci USA. 2016;113:15138–43 10.1073/pnas.1619047114 PMC5206524 27956642

[25] Zhang C, Tang Y, Tang S. et al. An inducible CRISPR activation tool for accelerating plant regeneration. Plant Commun. 2024;5:100823 10.1016/j.xplc.2024.100823 PMC11121170 38243597

[26] Liu X, Deng M, Shi B. et al. Distinct roles of H3K27me3 and H3K36me3 in vernalization response, maintenance, and resetting in winter wheat. Sci China Life Sci. 2024;67:2251–66 10.1007/s11427-024-2664-0 38987431

[27] Wang Z, Xia A, Wang Q. et al. Natural polymorphisms in *ZMET2* encoding a DNA methyltransferase modulate the number of husk layers in maize. Plant Physiol. 2024;195:2129–42 10.1093/plphys/kiae113 PMC11213254 38431291

[28] Khan IU, Ali A, Khan HA. et al. PWR/HDA9/ABI4 complex epigenetically regulates ABA dependent drought stress tolerance in *Arabidopsis*. Front Plant Sci. 2020;11:623 10.3389/fpls.2020.00623 PMC7266079 32528497

[29] Zheng L, Yu Y, Zheng Y. et al. Long small RNA76113 targets CYCLIC NUCLEOTIDE-GATED ION CHANNEL 5 to repress disease resistance in rice. Plant Physiol. 2024;194:1889–905 10.1093/plphys/kiad599 PMC10904327 37949839

[30] Ng DW, Miller M, Yu HH. et al. A role for CHH methylation in the parent-of-origin effect on altered circadian rhythms and biomass heterosis in *Arabidopsis* intraspecific hybrids. Plant Cell. 2014;26:2430–40 10.1105/tpc.113.115980 PMC4114943 24894042

[31] Chen Y, Li D, Lang Z. et al. An emerging role beyond genetics: DNA methylation in horticultural quality shaping. Innov Life. 2024;2:100050

[32] Liu S, Zhang Q, Kollie L. et al. Molecular networks of secondary metabolism accumulation in plants: current understanding and future challenges. Ind Crop Prod. 2023;201:116901

[33] Li T, Huang J, Wang G. et al. Regulatory roles of RNA modifications in plant development and fruit ripening. aBIOTECH. 2025;6:472–88 10.1007/s42994-025-00240-5 PMC12454768 40994446

[34] Ji Y, Wang A. Recent advances in epigenetic triggering of climacteric fruit ripening. Plant Physiol. 2023;192:1711–7 10.1093/plphys/kiad206 PMC10315304 37002826

[35] An C, Liu Z, Pan X. et al. Effect of histone modifications on fruit ripening. Physiol Plant. 2024;176:e14639 10.1111/ppl.14639 39618143

[36] Li X, Zhang Z, Luo X. et al. The exploration of *N^6^*-deoxyadenosine methylation in mammalian genomes. Protein Cell. 2021;12:756–68 10.1007/s13238-021-00866-3 PMC8464638 34405377

[37] Zhang H, Gong Z, Zhu JK. Active DNA demethylation in plants: 20 years of discovery and beyond. J Integr Plant Biol. 2022;64:2217–39 10.1111/jipb.13423 36478523

[38] Liang Z, Shen L, Cui X. et al. DNA *N^6^*-adenine methylation in *Arabidopsis thaliana*. Dev Cell. 2018;45:406–416.e3 10.1016/j.devcel.2018.03.012 29656930

[39] Zhang X, Jia Q, Wang W. et al. Microrchidia ATPases and DNA 6mA demethylase ALKBH1 act antagonistically on PRC2 to control chromatin structure and stress tolerance. Nat Plants. 2025;11:1591–607 10.1038/s41477-025-02048-z 40817150

[40] Guo W, Le L, Zhang D. Heat stress responses mediated by *N^6^*-methyladenine DNA methylation in maize. Cell Rep. 2025;44:116058 10.1016/j.celrep.2025.116058 40728929

[41] Bewick AJ, Niederhuth CE, Ji L. et al. The evolution of CHROMOMETHYLASES and gene body DNA methylation in plants. Genome Biol. 2017;18:65 10.1186/s13059-017-1195-1 PMC5410703 28457232

[42] Wassenegger M, Heimes S, Riedel L. et al. RNA-directed *de novo* methylation of genomic sequences in plants. Cell. 1994;76:567–76 10.1016/0092-8674(94)90119-8 8313476

[43] Zhong X, Du J, Hale CJ. et al. Molecular mechanism of action of plant DRM *de novo* DNA methyltransferases. Cell. 2014;157:1050–60 10.1016/j.cell.2014.03.056 PMC4123750 24855943

[44] Cuerda-Gil D, Slotkin RK. Non-canonical RNA-directed DNA methylation. Nat Plants. 2016;2:16163 10.1038/nplants.2016.163 27808230

[45] Zhang H, Zhu JK. Epigenetic gene regulation in plants and its potential applications in crop improvement. Nat Rev Mol Cell Biol. 2025;26:51–67 10.1038/s41580-024-00769-1 39192154

[46] Stroud H, Greenberg MV, Feng S. et al. Comprehensive analysis of silencing mutants reveals complex regulation of the *Arabidopsis* methylome. Cell. 2013;152:352–64 10.1016/j.cell.2012.10.054 PMC3597350 23313553

[47] Stroud H, Do T, Du J. et al. Non-CG methylation patterns shape the epigenetic landscape in *Arabidopsis*. Nat Struct Mol Biol. 2014;21:64–72 10.1038/nsmb.2735 PMC4103798 24336224

[48] Edger PP, Poorten TJ, VanBuren R. et al. Origin and evolution of the octoploid strawberry genome. Nat Genet. 2019;51:541–7 10.1038/s41588-019-0356-4 PMC6882729 30804557

[49] Sun Y, Yang X, Wu R. et al. DNA methylation controlling abscisic acid catabolism responds to light to mediate strawberry fruit ripening. J Integr Plant Biol. 2024;66:1718–34 10.1111/jipb.13681 38896078

[50] Li YM, Zhang HX, Tang XS. et al. Abscisic acid induces DNA methylation alteration in genes related to berry ripening and stress response in grape (*Vitis vinifera* L). J Agric Food Chem. 2024;72:15027–39 10.1021/acs.jafc.4c02303 38886897

[51] Huang H, Liu R, Niu Q. et al. Global increase in DNA methylation during orange fruit development and ripening. Proc Natl Acad Sci USA. 2019;116:1430–6 10.1073/pnas.1815441116 PMC6347674 30635417

[52] Wei TL, Wan YT, Liu HN. et al. CHH hypermethylation contributes to the early ripening of grapes revealed by DNA methylome landscape of 'Kyoho' and its bud mutant. Hortic Res. 2025;12:uhae285 10.1093/hr/uhae285 PMC11764089 39866961

[53] Kong J, Ma Y, Huang H. et al. The grape berry methylome reveals tissue-specific features associated with metabolism in ripening. Hortic Res. 2025;12:uhaf238 10.1093/hr/uhaf238 PMC12682070 41362890

[54] Cheng J, Niu Q, Zhang B. et al. Downregulation of RdDM during strawberry fruit ripening. Genome Biol. 2018;19:212 10.1186/s13059-018-1587-x PMC6280534 30514401

[55] Ooi SK, Bestor TH. The colorful history of active DNA demethylation. Cell. 2008;133:1145–8 10.1016/j.cell.2008.06.009 18585349

[56] Gong Z, Morales-Ruiz T, Ariza RR. et al. ROS1, a repressor of transcriptional gene silencing in Arabidopsis, encodes a DNA glycosylase/lyase. Cell. 2002;111:803–14 10.1016/s0092-8674(02)01133-9 12526807

[57] Du X, Yang Z, Xie G. et al. Molecular basis of the plant ROS1-mediated active DNA demethylation. Nat Plants. 2023;9:271–9 10.1038/s41477-022-01322-8 36624257

[58] Cao X, Li X, Su Y. et al. Transcription factor PpNAC1 and DNA demethylase PpDML1 synergistically regulate peach fruit ripening. Plant Physiol. 2024;194:2049–68 10.1093/plphys/kiad627 37992120

[59] Niu Q, Xu Y, Huang H. et al. Two transcription factors play critical roles in mediating epigenetic regulation of fruit ripening in tomato. Proc Natl Acad Sci USA. 2025;122:e2422798122 10.1073/pnas.2422798122 PMC12012504 40203043

[60] Ding X, Liu X, Jiang G. et al. SlJMJ7 orchestrates tomato fruit ripening via crosstalk between H3K4me3 and DML2-mediated DNA demethylation. New Phytol. 2022;233:1202–19 10.1111/nph.17838 34729792

[61] Liu R, How-Kit A, Stammitti L. et al. A DEMETER-like DNA demethylase governs tomato fruit ripening. Proc Natl Acad Sci USA. 2015;112:10804–9 10.1073/pnas.1503362112 PMC4553810 26261318

[62] Lang Z, Wang Y, Tang K. et al. Critical roles of DNA demethylation in the activation of ripening-induced genes and inhibition of ripening-repressed genes in tomato fruit. Proc Natl Acad Sci USA. 2017;114:E4511–9 10.1073/pnas.1705233114 PMC5465898 28507144

[63] Zhou L, Gao G, Li X. et al. The pivotal ripening gene SlDML2 participates in regulating disease resistance in tomato. Plant Biotechnol J. 2023;21:2291–306 10.1111/pbi.14130 PMC10579708 37466912

[64] Song B, Yu J, Li X. et al. Increased DNA methylation contributes to the early ripening of pear fruits during domestication and improvement. Genome Biol. 2024;25:87 10.1186/s13059-024-03220-y PMC10996114 38581061

[65] Gu C, Pei MS, Guo ZH. et al. Multi-omics provide insights into the regulation of DNA methylation in pear fruit metabolism. Genome Biol. 2024;25:70 10.1186/s13059-024-03200-2 PMC10938805 38486226

[66] Wang J, Wang J, Li Y. et al. Epigenomic mechanism regulating the quality and ripeness of apple fruit with differing harvest maturity. Physiol Plant. 2024;176:e14278 10.1111/ppl.14278 38644530

[67] Kong J, Xu X, Carbonell-Bejerano P. et al. Epiallelic variation at the transcription factor *MYBA1* promoter contributes to color differences of berry flesh in grapevine. Plant Physiol. 2025;198:kiaf340 10.1093/plphys/kiaf340 40795055

[68] Xu J, Xiong L, Yao JL. et al. Hypermethylation in the promoter regions of flavonoid pathway genes is associated with skin color fading during 'Daihong' apple fruit development. Hortic Res. 2024;11:uhae031 10.1093/hr/uhae031 PMC10933707 38481937

[69] Zhang P, Zhao Q, Song Y. et al. Identification of key genes controlling anthocyanin biosynthesis in the fruits of a bud variety of Tarocco blood-orange. BMC Plant Biol. 2025;25:230 10.1186/s12870-025-06212-7 PMC11841362 39979800

[70] Zhu YC, Zhang B, Allan AC. et al. DNA demethylation is involved in the regulation of temperature-dependent anthocyanin accumulation in peach. Plant J. 2020;102:965–76 10.1111/tpj.14680 31923329

[71] Yu H, Zhang C, Lu C. et al. The lemon genome and DNA methylome unveil epigenetic regulation of citric acid biosynthesis during fruit development. Hortic Res. 2024;11:uhae005 10.1093/hr/uhae005 PMC10923643 38464476

[72] Wang Y, Zeng Y, Wang Y. et al. Haplotype-resolved genome and multi-omics landscape reveal an epigenetic regulation of citric acid accumulation in lemon. Plant Physiol. 2025;199:kiaf400 10.1093/plphys/kiaf400 40913443

[73] Bai F, Wu M, Huang W. et al. Removal of toxic steroidal glycoalkaloids and bitterness in tomato is controlled by a complex epigenetic and genetic network. Sci Adv. 2025;11:eads9601 10.1126/sciadv.ads9601 PMC11837996 39970214

[74] Guo H, Cao P, Wang C. et al. Population analysis reveals the roles of DNA methylation in tomato domestication and metabolic diversity. Sci China Life Sci. 2023;66:1888–902 10.1007/s11427-022-2299-5 36971992

[75] Black JC, Van Rechem C, Whetstine JR. Histone lysine methylation dynamics: establishment, regulation, and biological impact. Mol Cell. 2012;48:491–507 10.1016/j.molcel.2012.11.006 PMC3861058 23200123

[76] Strahl BD, Allis CD. The language of covalent histone modifications. Nature. 2000;403:41–5 10.1038/47412 10638745

[77] Zhang P, He Y, Huang S. Unlocking epigenetic breeding potential in tomato and potato. aBIOTECH. 2024;5:507–18 10.1007/s42994-024-00184-2 PMC11624185 39650134

[78] Song Y, Chang Z, Feng Y. et al. Whole-genome landscape of histone H3K4me3 modification during sperm cell lineage development in tomato. BMC Plant Biol. 2024;24:610 10.1186/s12870-024-05318-8 PMC11210149 38926660

[79] Jaiswal N, Liao CJ, Hewavidana AI. et al. GCN5-related histone acetyltransferase HOOKLESS2 regulates fungal resistance and growth in tomato. New Phytol. 2025;246:1217–35 10.1111/nph.70025 PMC11982796 40022479

[80] Zhang X, Zhang S, Wang S. et al. The ETHYLENE RESPONSE FACTOR6-*GRETCHEN HAGEN3.5* module regulates rooting and heat tolerance in *Dimocarpus longan*. Plant Physiol. 2025;197:kiaf096 10.1093/plphys/kiaf096 PMC11950727 40106655

[81] Chen D, Ma H, Hong H. et al. Regulation of transcription by a protein methyltransferase. Science. 1999;284:2174–7 10.1126/science.284.5423.2174 10381882

[82] Rea S, Eisenhaber F, O'Carroll D. et al. Regulation of chromatin structure by site-specific histone H3 methyltransferases. Nature. 2000;406:593–9 10.1038/35020506 10949293

[83] Shi Y, Lan F, Matson C. et al. Histone demethylation mediated by the nuclear amine oxidase homolog LSD1. Cell. 2004;119:941–53 10.1016/j.cell.2004.12.012 15620353

[84] Van Rechem C, Whetstine JR. Examining the impact of gene variants on histone lysine methylation. Biochim Biophys Acta. 2014;1839:1463–76 10.1016/j.bbagrm.2014.05.014 PMC4752941 24859469

[85] Li Z, Jiang G, Liu X. et al. Histone demethylase SlJMJ6 promotes fruit ripening by removing H3K27 methylation of ripening-related genes in tomato. New Phytol. 2020;227:1138–56 10.1111/nph.16590 32255501

[86] Li Z, Zeng J, Zhou Y. et al. Histone H3K27 demethylase SlJMJ3 modulates fruit ripening in tomato. Plant Physiol. 2024;195:2727–42 10.1093/plphys/kiae233 38669310

[87] Zeng J, Li Z, Ding X. et al. The H3R2me2a demethylase JMJ10 regulates tomato fruit size through its interaction with the transcription factor BZR1.3. Plant Cell. 2025;37:koaf251 10.1093/plcell/koaf251 41100692

[88] Yi-Zhe Cheng YZ, Sun YD, Pei MS. et al. Transcription factor VviAGL6a regulates fruit ripening by directly activating grape *VviJMJ21*. Sci Hortic. 2024;336:113396

[89] Zuo D, Yang L, Li R. et al. Histone H3K4 demethyltransferase VvJMJ14 promotes H_2_O_2_ inducing fruit ripening in grape. Plant J. 2025;124:e70642 10.1111/tpj.70642 41389676

[90] Allfrey VG, Faulkner R, Mirsky AE. Acetylation and methylation of histones and their possible role in the regulation of RNA synthesis. Proc Natl Acad Sci USA. 1964;51:786–94 10.1073/pnas.51.5.786 PMC300163 14172992

[91] Grunstein M . Histone acetylation in chromatin structure and transcription. Nature. 1997;389:349–52 10.1038/38664 9311776

[92] Deng H, Chen Y, Liu Z. et al. SlERF.F12 modulates the transition to ripening in tomato fruit by recruiting the co-repressor TOPLESS and histone deacetylases to repress key ripening genes. Plant Cell. 2022;34:1250–72 10.1093/plcell/koac025 PMC8972228 35099538

[93] He X, Wu Y, Shu P. et al. SlPLT6 controls ripening initiation and quality traits through modulation of histone acetylation and methylation in tomato. Proc Natl Acad Sci USA. 2025;122:e2503732122 10.1073/pnas.2503732122 PMC12184660 40493199

[94] Han YC, Kuang JF, Chen JY. et al. Banana transcription factor MaERF11 recruits histone deacetylase MaHDA1 and represses the expression of *MaACO1* and expansins during fruit ripening. Plant Physiol. 2016;171:1070–84 10.1104/pp.16.00301 PMC4902611 27208241

[95] Hu Y, Han Z, Wang T. et al. Ethylene response factor MdERF4 and histone deacetylase MdHDA19 suppress apple fruit ripening through histone deacetylation of ripening-related genes. Plant Physiol. 2022;188:2166–81 10.1093/plphys/kiac016 PMC8968277 35088866

[96] Jia H, Zuo Q, Sadeghnezhad E. et al. HDAC19 recruits ERF4 to the *MYB5a* promoter and diminishes anthocyanin accumulation during grape ripening. Plant J. 2023;113:127–44 10.1111/tpj.16040 36423230

[97] Pan Q, Guo S, Ding J. et al. Dynamic histone modification signatures coordinate developmental programs in strawberry fruit ripening. Hortic Res. 2024;11:uhae158 10.1093/hr/uhae158 PMC11298626 39108587

[98] Wang L, Lin Y, Hou G. et al. A histone deacetylase, *FaSRT1-2*, plays multiple roles in regulating fruit ripening, plant growth and stresses resistance of cultivated strawberry. Plant Cell Environ. 2024;47:2258–73 10.1111/pce.14885 38482979

[99] Yang M, Song C, He X. et al. The new function of *FaSRT2-1* protein in energy metabolism: promoting strawberry fruit quality and ripening. Int J Biol Macromol. 2024;281:136199 10.1016/j.ijbiomac.2024.136199 39366613

[100] Mukhopadhyay U, Levantovsky S, Carusone TM. et al. A ubiquitin-specific, proximity-based labeling approach for the identification of ubiquitin ligase substrates. Sci Adv. 2024;10:eadp3000 10.1126/sciadv.adp3000 PMC11313854 39121224

[101] Wang H, Wang L, Erdjument-Bromage H. et al. Role of histone H2A ubiquitination in polycomb silencing. Nature. 2004;431:873–8 10.1038/nature02985 15386022

[102] Jiang M, Yang Y, Wei W. et al. Interaction of MaERF11 with the E3 ubiquitin ligase MaRFA1 is involved in the regulation of banana starch degradation during postharvest ripening. Hortic Plant J. 2025;11:608–18

[103] Wang Y, Wang W, Cai J. et al. Tomato nuclear proteome reveals the involvement of specific E2 ubiquitin-conjugating enzymes in fruit ripening. Genome Biol. 2014;15:548 10.1186/s13059-014-0548-2 PMC4269173 25464976

[104] Jia H, Jia H, Lu S. et al. DNA and histone methylation regulates different types of fruit ripening by transcriptome and proteome analyses. J Agric Food Chem. 2022;70:3541–56 10.1021/acs.jafc.1c06391 35266388

[105] Xu C, Li R, Chen X. et al. Tomato ripening regulator SlSAD8 disturbs nuclear gene transcription and chloroplast-associated protein degradation. Nat Plants. 2025;11:2230–9 10.1038/s41477-025-02134-2 41107469

[106] Pei Y, He X, Xue Q. et al. The bifunctional transcription factor DEAR1 oppositely regulates chlorophyll biosynthesis and degradation in tomato fruits. Plant Cell. 2025;37:koaf167 10.1093/plcell/koaf167 40577586

[107] He X, Liu K, Wu Y. et al. A transcriptional cascade mediated by two APETALA2 family members orchestrates carotenoid biosynthesis in tomato. J Integr Plant Biol. 2024;66:1227–41 10.1111/jipb.13650 38546046

[108] Huang B, Li Y, Jia K. et al. The MdMYB44-MdTPR1 repressive complex inhibits *MdCCD4* and *MdCYP97A3* expression through histone deacetylation to regulate carotenoid biosynthesis in apple. Plant J. 2024;119:540–56 10.1111/tpj.16782 38662911

[109] Ni J, Wang S, Yu W. et al. The ethylene-responsive transcription factor PpERF9 represses *PpRAP2.4* and *PpMYB114* via histone deacetylation to inhibit anthocyanin biosynthesis in pear. Plant Cell. 2023;35:2271–92 10.1093/plcell/koad077 PMC10226596 36916511

[110] Wei C, Yang H, Ye B. et al. Ubiquitination of the PpMADS2 transcription factor controls linalool production during UV-B irradiation in detached peach fruit. Plant Physiol. 2025;198:kiaf159 10.1093/plphys/kiaf159 40273302

[111] Sonawane PD, Gharat SA, Jozwiak A. et al. A BAHD-type acyltransferase concludes the biosynthetic pathway of non-bitter glycoalkaloids in ripe tomato fruit. Nat Commun. 2023;14:4540 10.1038/s41467-023-40092-5 PMC10374582 37500644

[112] Lin X, Shi Y, Liu S. et al. Genome-wide identification of the histone acetyltransferase gene family in *Citrus clementina* and its potential roles in citrate metabolism. Food Qual Saf. 2024;8:1–11

[113] Zhang M, Tan FQ, Fan YJ. et al. Acetylome reprograming participates in the establishment of fruit metabolism during polyploidization in citrus. Plant Physiol. 2022;190:2519–38 10.1093/plphys/kiac442 PMC9706433 36135821

[114] Motorin Y, Helm M. RNA nucleotide methylation: 2021 update. Wiley Interdiscip Rev: RNA. 2022;13:e1691 10.1002/wrna.1691 34913259

[115] Meyer KD, Saletore Y, Zumbo P. et al. Comprehensive analysis of mRNA methylation reveals enrichment in 3' UTRs and near stop codons. Cell. 2012;149:1635–46 10.1016/j.cell.2012.05.003 PMC3383396 22608085

[116] Zhang J, Wu L, Mu L. et al. Evolution and post-transcriptional regulation insights of m^6^A writers, erasers, and readers in plant epitranscriptome. Plant J. 2024;120:505–25 10.1111/tpj.16996 39167634

[117] Yan X, Pei K, Guan Z. et al. AI-empowered integrative structural characterization of m^6^A methyltransferase complex. Cell Res. 2022;32:1124–7 10.1038/s41422-022-00741-8 PMC9715571 36357785

[118] Liang Z, Riaz A, Chachar S. et al. Epigenetic modifications of mRNA and DNA in plants. Mol Plant. 2020;13:14–30 10.1016/j.molp.2019.12.007 31863849

[119] Yue H, Nie X, Yan Z. et al. *N^6^*-methyladenosine regulatory machinery in plants: composition, function and evolution. Plant Biotechnol J. 2019;17:1194–208 10.1111/pbi.13149 PMC6576107 31070865

[120] Zhou L, Gao G, Tang R. et al. m^6^A-mediated regulation of crop development and stress responses. Plant Biotechnol J. 2022;20:1447–55 10.1111/pbi.13792 PMC9342612 35178842

[121] Shi Y, Yang S, Pei T. et al. The m^6^A writers, readers, and erasers regulate plant development and respond to biotic/abiotic stresses. Epigenetics Insights. 2025;18:e008

[122] Fu D, Wang H, Jiang B. Molecular mechanisms and crop improvement potential of RNA *N^6^*-methyladenosine in plants. aBIOTECH. 2025;6:489–509 10.1007/s42994-025-00228-1 PMC12454794 40994448

[123] Shan C, Dong K, Wen D. et al. A review of m^6^A modification in plant development and potential quality improvement. Int J Biol Macromol. 2025;308:142597 10.1016/j.ijbiomac.2025.142597 40157682

[124] Hu J, Cai J, Umme A. et al. Unique features of mRNA m^6^A methylomes during expansion of tomato (*Solanum lycopersicum*) fruits. Plant Physiol. 2022;188:2215–27 10.1093/plphys/kiab509 PMC8968293 34730815

[125] Zhou L, Gao G, Tang R. et al. Redox modification of m^6^A demethylase SlALKBH2 in tomato regulates fruit ripening. Nat Plants. 2025;11:218–33 10.1038/s41477-024-01893-8 39794492

[126] Zhou L, Tian S, Qin G. RNA methylomes reveal the m^6^A-mediated regulation of DNA demethylase gene *SlDML2* in tomato fruit ripening. Genome Biol. 2019;20:156 10.1186/s13059-019-1771-7 PMC6683476 31387610

[127] Gao Y, Bian H, Wang H. et al. The m^6^A reader SlYTH1 regulates flavor-related volatiles biosynthesis via affecting mRNA stability and translation in tomato fruit. Plant Physiol. 2025;198:kiaf245 10.1093/plphys/kiaf245 40479511

[128] Bian H, Song P, Gao Y. et al. The m^6^A reader SlYTH2 negatively regulates tomato fruit aroma by impeding the translation process. Proc Natl Acad Sci USA. 2024;121:e2405100121 10.1073/pnas.2405100121 PMC11253005 38950372

[129] Su D, Shu P, Hu N. et al. Dynamic m^6^A mRNA methylation reveals the involvement of AcALKBH10 in ripening-related quality regulation in kiwifruit. New Phytol. 2024;243:2265–78 10.1111/nph.20008 39056285

[130] Xin T, Zhang Z, Zhang Y. et al. Recessive epistasis of a synonymous mutation confers cucumber domestication through epitranscriptomic regulation. Cell. 2025;188:4517–4529.e15 10.1016/j.cell.2025.06.007 40602402

[131] Zhou L, Tang R, Li X. et al. *N^6^*-methyladenosine RNA modification regulates strawberry fruit ripening in an ABA-dependent manner. Genome Biol. 2021;22:168 10.1186/s13059-021-02385-0 PMC8173835 34078442

[132] Tang R, Duan X, Zhou L. et al. The FvABF3-FvALKBH10B-FvSEP3 cascade regulates fruit ripening in strawberry. Nat Commun. 2024;15:10912 10.1038/s41467-024-55294-8 PMC11685502 39738062

[133] Dominissini D, Nachtergaele S, Moshitch-Moshkovitz S. et al. The dynamic *N^1^*-methyladenosine methylome in eukaryotic messenger RNA. Nature. 2016;530:441–6 10.1038/nature16998 PMC4842015 26863196

[134] Ma L, Zuo J, Bai C. et al. The dynamic *N^1^*-methyladenosine RNA methylation provides insights into the tomato fruit ripening. Plant J. 2024;120:2014–30 10.1111/tpj.17095 39495773

[135] Yang X, Yang Y, Sun BF. et al. 5-methylcytosine promotes mRNA export — NSUN2 as the methyltransferase and ALYREF as an m^5^C reader. Cell Res. 2017;27:606–25 10.1038/cr.2017.55 PMC5594206 28418038

[136] Amort T, Rieder D, Wille A. et al. Distinct 5-methylcytosine profiles in poly(a) RNA from mouse embryonic stem cells and brain. Genome Biol. 2017;18:1 10.1186/s13059-016-1139-1 PMC5225599 28077169

[137] David R, Burgess A, Parker B. et al. Transcriptome-wide mapping of RNA 5-methylcytosine in *Arabidopsis* mRNAs and noncoding RNAs. Plant Cell. 2017;29:445–60 10.1105/tpc.16.00751 PMC5385953 28062751

[138] Guo S, Zheng Y, Meng D. et al. DNA and coding/non-coding RNA methylation analysis provide insights into tomato fruit ripening. Plant J. 2022;112:399–413 10.1111/tpj.15951 36004545

[139] Ito S, Horikawa S, Suzuki T. et al. Human NAT10 is an ATP-dependent RNA acetyltransferase responsible for *N^4^*-acetylcytidine formation in 18S ribosomal RNA (rRNA). J Biol Chem. 2014;289:35724–30 10.1074/jbc.C114.602698 PMC4276842 25411247

[140] Li B, Li D, Cai L. et al. Transcriptome-wide profiling of RNA *N^4^*-cytidine acetylation in *Arabidopsis thaliana* and *Oryza sativa*. Mol Plant. 2023;16:1082–98 10.1016/j.molp.2023.04.009 37073130

[141] Ma L, Zheng Y, Zhou Z. et al. Dissection of mRNA ac^4^C acetylation modifications in AC and Nr fruits: insights into the regulation of fruit ripening by ethylene. Mol Hortic. 2024;4:5 10.1186/s43897-024-00082-7 PMC10875755 38369544

[142] Suraweera A, O'Byrne KJ, Richard DJ. Epigenetic drugs in cancer therapy. Cancer Metastasis Rev. 2025;44:37 10.1007/s10555-025-10253-7 PMC11865116 40011240

[143] Zhong S, Fei Z, Chen YR. et al. Single-base resolution methylomes of tomato fruit development reveal epigenome modifications associated with ripening. Nat Biotechnol. 2013;31:154–9 10.1038/nbt.2462 23354102

[144] Wang Y, Wang Y, Li F. et al. Methylation profiling of biosynthetic genes reveals the role of D-galacturonic acid reductase in ascorbic acid accumulation in tomato fruit. Plant Growth Regul. 2022;98:281–8

[145] Yang L, Liu R, Meng Y. et al. Fruit phenotype analysis of *SlSAHH2*-CRISPR tomato and methylation mechanism of *SlSAHH2* promoting fruit ripening. J Agric Food Chem. 2025;73:18691–705 10.1021/acs.jafc.5c05207 40682549

[146] Guo DL, Qiong L, Zhao HL. et al. The variation of berry development and DNA methylation after treatment with 5-azaC on 'Kyoho' grape. Sci Hortic. 2019;246:265–71

[147] Guo DL, Li Q, Ji XR. et al. Transcriptome profiling of 'Kyoho' grape at different stages of berry development following 5-azaC treatment. BMC Genome. 2019;20:825 10.1186/s12864-019-6204-1 PMC6839162 31703618

[148] Jia H, Su Z, Ren Y. et al. Exploration of 5-azacytidine and trichostatin a in the modulation of postharvest quality of 'Shine Muscat' grape berries. Postharvest Biol Technol. 2024;209:112719

[149] Tang W, Li F, Yang D. et al. Characterization of lysine acetyltransferase and deacetylase genes and their roles in anthocyanin biosynthesis in grape. Hortic Adv. 2025;3:2

[150] Zheng Y, Chen X, Chen W. et al. Inhibition of DNA methylation modification regulates endogenous GABA, proline and AsA-GSH metabolism and enhances antioxidant capacity to improve chilling tolerance in postharvest kiwifruit. Postharvest Biol Technol. 2025;230:113812

[151] Lee JH, Choy ML, Ngo L. et al. Histone deacetylase inhibitor induces DNA damage, which normal but not transformed cells can repair. Proc Natl Acad Sci USA. 2010;107:14639–44 10.1073/pnas.1008522107 PMC2930422 20679231

[152] Bertoli R, Cao D, Tuckey O. et al. 5-azacytidine and decitabine induce C > G transversions in both murine and human cells. Nat Commun. 2025;39:2112–24 10.1038/s41375-025-02670-y PMC12380594 40681875

[153] Li H, Chen Z, Zhu W. et al. The MaNAP1-MaMADS1 transcription factor module mediates ethylene-regulated peel softening and ripening in banana. Plant Cell. 2024;37:koae282 10.1093/plcell/koae282 PMC11663587 39422253

[154] Tao J, Wang Y, Fernie AR. et al. Transcriptomic and epigenetic signatures of tomato fruit after postharvest UV-C irradiation are associated with the maintenance of fruit quality. Plant Biotechnol J. 2025;23:4500–2 10.1111/pbi.70210 PMC12483979 41028991

[155] Xi H, Tong L, Xu C. et al. Identification and characterization of genes related to m^6^A modification in kiwifruit using RNA-seq and ATAC-seq. Hortic Plant J. 2024;10:1121–33

[156] Urich MA, Nery JR, Lister R. et al. MethylC-seq library preparation for base-resolution whole-genome bisulfite sequencing. Nat Protoc. 2015;10:475–83 10.1038/nprot.2014.114 PMC4465251 25692984

[157] Zhou M, Palanca AMS, Law JA. Locus-specific control of the de novo DNA methylation pathway in *Arabidopsis* by the CLASSY family. Nat Genet. 2018;50:865–73 10.1038/s41588-018-0115-y PMC6317521 29736015

[158] Nishio H, Nagano AJ, Ito T. et al. Seasonal plasticity and diel stability of H3K27me3 in natural fluctuating environments. Nat Plants. 2020;6:1091–7 10.1038/s41477-020-00757-1 32868888

